# Beat-locked ATP microdomains in the sinoatrial node map a Ca^2+^-timed energetic hierarchy and regional pacemaker roles

**DOI:** 10.1085/jgp.202513874

**Published:** 2026-03-31

**Authors:** Manuel F. Muñoz, Collin Matsumoto, Paula Rhana, Declan Manning, Geoanna M. Bautista, Daniel M. Collier, L. Fernando Santana

**Affiliations:** 1 Department of Physiology and Membrane Biology, School of Medicine, University of California, Davis, Davis, CA, USA; 2 Department of Pediatrics, School of Medicine, University of California, Davis, Davis, CA, USA; 3 Department of Pharmaceutical Sciences, College of Pharmacy, The University of Tennessee Health Science Center, Memphis, TN, USA

## Abstract

Pacemaker cells of the sinoatrial (SA) node fire spontaneously and continuously under normal conditions, sustaining a high energetic cost with every heartbeat. How they meet this demand has remained poorly understood. Using genetically encoded fluorescent sensors targeted to the cytosol and mitochondria, we tracked ATP in real time within myocytes of the mouse SA node. Rather than maintaining a steady energy reserve, these cells produce and consume ATP in precise, beat-by-beat bursts in both compartments, synchronized to each Ca^2+^ transient that triggers a heartbeat—a just-in-time energetic strategy. Not all pacemaker cells operate equally. Cells in the superior SA node, better supplied by blood vessels and rich in mitochondria, produce ATP more efficiently with each beat—a high-gain phenotype. Cells in the inferior node, more sparsely vascularized, operate in a lower gain or energy-deficit mode. These distinct energetic profiles set limits on the firing frequencies each cell can sustain, determining which cells drive fast rates and which support stable rhythm across a broader frequency range. Blocking Ca^2+^ transfer into mitochondria or impairing ATP export abolished beat-locked energy signals, consistent with mitochondrial Ca^2+^ uniporter–ANT machinery coupling Ca^2+^ release to ATP fluctuations. Strikingly, disrupting mitochondrial energy production rendered pacemaker cells electrically silent, suggesting that mitochondrial ATP synthesis is essential for excitability. Together, these findings suggest that beat-locked, just-in-time ATP generation is integral to cardiac pacemaking itself, with local blood supply, mitochondrial capacity, and Ca^2+^ signaling shaping which cells preferentially set heart rate and which support stable firing across a broader frequency range.

## Teaser

Beat-locked cytosolic and mitochondrial ATP transients in SA node myocytes sort into high-gain, low-gain, or consumption-dominant modes aligned with superior–inferior vascular–mitochondrial gradients. This energetic hierarchy lets high-gain cells set fast rates, while low-gain/dip cells stabilize slow rhythms, broadening operating range but capping maximal bandwidth.

## Introduction

The heartbeat originates in the sinoatrial (SA) node, a crescent-shaped pacemaker region embedded at the junction of the superior vena cava and right atrium. Within the node, clusters of pacemaker myocytes fire spontaneous action potentials that propagate through gap junctions to neighboring SA node myocytes and, ultimately, the surrounding myocardium.

Pacemaking emerges from the concerted operation of a “membrane clock,” composed of voltage-gated ion channels, including hyperpolarization-activated, cyclic nucleotide-gated channels (HCN2/HCN4) and Ca^2+^ channels (Ca_V_3.1, Ca_V_1.3, and Ca_V_1.2) (Brown and Difrancesco, 1980; DiFrancesco, 1986; Hüser et al., 2000; Mangoni et al., 2006), and a “Ca^2+^ clock,” timed by stochastic, diastolic Ca^2+^ sparks from the sarcoplasmic reticulum (SR) that activate inward Na^+^/Ca^2+^-exchanger current (Bogdanov et al., 2001). These coupled oscillators depolarize the membrane to threshold, trigger a cell-wide, global intracellular Ca^2+^ ([Ca^2+^]_i_) transient, and, after repolarization by K^+^ currents, reset to begin the next diastolic depolarization.

A defining feature of the SA node is its functional and anatomical heterogeneity (Boyett et al., 2000). In the mouse SA node, for example, inferior regions fire more slowly and are more sparsely vascularized (Grainger et al., 2021; Manning et al., 2025). This spatial organization creates subpopulations with distinct intrinsic firing rates, metabolic throughput, and coupling, suggesting that network rhythm could arise through at least two non-exclusive mechanisms: classical entrainment (Jalife, 1984; Anumonwo et al., 1991)—where electrotonic coupling pulls disparate oscillators toward a common period—and stochastic resonance—where variability from a noisier subpopulation can enhance sensitivity and bandwidth (Maltsev et al., 2011; Clancy and Santana, 2020; Grainger et al., 2021; Guarina et al., 2022; Okamura et al., 2024, *Preprint*).

Each beat carries a steep energetic cost. Adenosine triphosphate (ATP) hydrolysis is required to maintain Na^+^ and K^+^ gradients via the Na^+^/K^+^ ATPase, refill the SR with Ca^2+^ through the SR/endoplasmic reticulum Ca^2+^ ATPase (SERCA) pump, support cross-bridge cycling, and fuel the continual adenylate cyclase–mediated production of cAMP that tunes both clock components (Yaniv et al., 2013). Even under basal conditions, isolated mouse SA node myocytes consume more O_2_ than ventricular myocytes paced at 3 Hz, and their ATP turnover rises further during sympathetic stimulation (Yaniv et al., 2013). About 95% of this ATP is supplied by mitochondrial oxidative metabolism and the remaining ∼5% by glycolysis (Wisneski et al., 1990; Saddik and Lopaschuk, 1991; Zhou and Tian, 2018; Lopaschuk et al., 2021).

Classic work has established coupling between Ca^2+^ handling and ATP production, showing that Ca^2+^ entering the mitochondrial matrix via the mitochondrial Ca^2+^ uniporter (MCU) activates three Ca^2+^-sensitive dehydrogenases—pyruvate, isocitrate, and 2-oxoglutarate dehydrogenase—boosting NADH production and driving proton flux through the ATP5A-containing F_1_F_o_-ATP synthase (Denton et al., 1980; Denton and McCormack, 1990; McCormack et al., 1990; Kirichok et al., 2004; Garbincius and Elrod, 2022). In this process, mitochondrial efficiency is shaped not only by enzymatic capacity but also by organelle architecture and proximity to SR Ca^2+^-release sites. The tethering protein mitofusin-2 (Mfn2), located on the outer mitochondrial membrane, mediates physical and functional coupling between mitochondria and the junctional SR, facilitating rapid Ca^2+^ transfer and enhancing oxidative phosphorylation (Chen et al., 2012; Dorn et al., 2015). Mitochondrial ATP production is supplemented by glycolysis and is further supported by creatine and adenylate kinase reactions, which draw on phosphocreatine and ADP pools to regenerate ATP, with adenine nucleotide translocase (ANT) coupling these processes by exporting mitochondrial ATP in exchange for cytosolic ADP. These pathways act in concert to maintain ATP supply across varying workloads.

To match the relentless demand for ATP by SA node myocytes, the SA node artery (most commonly a branch of the right coronary artery) fans into an elaborate capillary network that perfuses the node during diastole when extravascular compression is lowest. High-resolution, cleared-tissue reconstructions show that capillary density is greatest and myocyte-to-vessel distance shortest in the superior SA node, whereas inferior regions are more sparsely vascularized (Grainger et al., 2021; Manning et al., 2025). These structural gradients correlate with intrinsic firing rate, suggesting that local energetic support sculpts pacemaker hierarchy. Yet, direct links between microvascular architecture, subcellular energetics, and pacemaking behavior have remained elusive.

Using genetically encoded fluorescent ATP sensors, we previously showed that under physiological workload, cytosolic ATP levels rise and fall with each [Ca^2+^]_i_ transient in ventricular myocytes (Rhana et al., 2024; Rhana et al., 2025, *Preprint*). This raised two questions for the SA node: Do spontaneously firing myocytes exhibit comparable beat-locked ATP dynamics, and do these dynamics differ between superior and inferior domains in a manner consistent with distinct pacemaking roles? Here, we use the term “bandwidth” descriptively to denote the range of firing frequencies over which a pacemaker cell can sustain stable oscillatory activity while meeting energetic demand.

To address these questions, we combined beat-resolved ATP imaging in the intact node and in isolated myocytes to (1) define two mitochondrial ATP modes time-locked to Ca^2+^ transients; (2) identify matched high- and low-gain cytosolic ATP phenotypes; (3) map these energetic phenotypes onto regional firing rate, mitochondrial volume, and capillary density; (4) demonstrate that Ca^2+^ release—via SR-to-mitochondrial Ca^2+^ transfer—acts as a master regulator that times oxidative ATP production to demand; and (5) reveal that mitochondrial uncoupling abolished stimulus-locked voltage signals and cytosolic ATP transients despite stepwise increases in field-stimulation intensity, indicating that oxidative phosphorylation is obligatory for SA node excitability and is not rescued by stronger electrical pacing. This hierarchy allocates pacemaking roles across the node and extends the emerging “paycheck-to-paycheck” model from ventricular myocytes (Rhana et al., 2024; Rhana et al., 2025, *Preprint*; Santana and Earley, 2026) to the SA node. Here, paycheck-to-paycheck denotes beat-locked ATP synthesis and use constrained by a local O_2_ ceiling—capillaries set the ceiling, mitochondria set the range of firing frequencies a cell can sustain while meeting energetic demand, and Ca^2+^ sets the timing—linking vascular architecture and mitochondrial organization to excitability and helping explain how distinct cell populations contribute to rate setting versus robustness across rates.

## Materials and methods

### Animals

Male wild-type C57BL/6J mice (The Jackson Laboratory) 8–12 wk old were used for all experiments in this study. Mice were euthanized with a single, intraperitoneal administration of a lethal dose of sodium pentobarbital (250 mg/kg). All procedures were performed in accordance with NIH guidelines and were approved by the Institutional Animal Care and Use Committee of the University of California, Davis, Davis, CA, USA.

### AAV-mediated delivery of ATP and voltage biosensors

Real-time visualization of intracellular ATP dynamics and changes in membrane voltage was enabled by systemically injecting mice with adeno-associated virus serotype 9 (AAV9) vectors encoding either the cytosolic ATP sensor iATPSnFR1.0 (cyto-iATP) (Lobas et al., 2019), the mitochondria-targeted variant iATPSnFR2.0 fused to HaloTag (mito-iATP) (Marvin et al., 2024), or the ultra-fast voltage sensor ASAP5 (Hao et al., 2024) at a titer of 4 × 10^12^ viral genomes per milliliter (vg/ml). The expression of these sensors was driven by the HCN4 promoter, restricting expression to SA node myocytes. AAV9 vectors (100 μl) were delivered via retro-orbital injection using a microsyringe into mice under isoflurane anesthesia (5% induction, 2% maintenance). Importantly, iATPSnFR1.0 and iATPSnFR2.0 sensors bind ATP with high specificity and exhibit negligible sensitivity to physiological fluctuations in free [Mg^2+^] (0–5 mM) (Lobas et al., 2019; Marvin et al., 2024). This insensitivity ensures that the observed fluorescence transients reflect genuine changes in ATP availability rather than artifacts arising from concurrent Mg^2+^ fluxes associated with ATP hydrolysis.

### Whole-mount immunolabeling of SA node tissue

Immunohistochemistry experiments on the SA node were performed as previously described (Grainger et al., 2021; Manning et al., 2025). Briefly, the SA node tissue was fixed in 4% paraformaldehyde in phosphate-buffered saline (PBS) for 30 min. Following fixation, the tissue was washed in PBS (3 × 5 min), transferred to a 15-ml tube, and washed in PBS for 12 h on a low-speed tube rotator. Tissues were dehydrated through a graded ethanol series (25, 50, 75, 95, and 100%), cleared in 20% dimethyl sulfoxide (DMSO) in ethanol for 2 h, and bleached overnight (12 h) in 6% hydrogen peroxide prepared in absolute ethanol. Samples were then rehydrated through a reversed ethanol gradient and washed in PBS (3 × 5 min). Tissues were permeabilized using 0.5% Triton X-100 in PBS (3 × 10 min) and blocked for 2 h at room temperature in PBS containing 5% normal donkey serum and 0.1% Triton X-100.

For immunolabeling, SA nodes were incubated for 48 h at 4°C with a goat anti-mouse CD31 primary antibody (1:50, AF3628; R&D Systems). After three 10-min PBS washes, tissues were incubated for 4 h at room temperature in the dark with donkey anti-goat Alexa Fluor 568 (1:1,000, A11057; Thermo Fisher Scientific) and/or anti-GFP Alexa Fluor 647 (1:1,000, A32985; Thermo Fisher Scientific) secondary antibodies. Following final PBS washes (3 × 10 min), tissues were incubated in a 1:4 solution of DMSO:PBS for 2 h, mounted in Aquamount (Thermo Fisher Scientific), coverslipped, and sealed for imaging.

### Confocal imaging and 3D segmentation of fixed tissue

Fixed SA node tissues were imaged using an Olympus FluoView 3000 inverted confocal microscope equipped with an APON 40× oil-immersion objective (numerical aperture [NA] = 1.3). To capture the entire SA node preparation, a mosaic of 3D image volumes (80–120 z-planes per volume) was acquired using Olympus FluoView software at a resolution of 0.5 µm/pixel across a 320-µm field of view. Subsequent image analysis and 3D segmentation were performed using Imaris 10 software (Oxford Instruments).

### Multiphoton imaging of [ATP]_i_, [ATP]_mito_, and FAD

Following confirmation of deep anesthesia, beating hearts were excised from animals expressing AAV9 constructs and transferred to a dish filled with a Tyrode III solution (37°C) consisting of 140 mM NaCl, 5.4 mM KCl, 1 mM MgCl_2_, 1.8 mM CaCl_2_, 5 mM HEPES, and 5.5 mM glucose (pH 7.4, adjusted with NaOH). Upon dissection, the SA node was pinned flat to a Sylgard-coated recording chamber containing Tyrode III supplemented with 10 μM blebbistatin (Sigma-Aldrich) for 1 h at 37°C.

Cyto-iATP and mito-iATP sensors in intact SA nodes were excited at 850 nm and imaged using an upright Olympus FVMPE-RS multiphoton microscope equipped with an XL Plan N 25× lens (NA = 1.05) in line-scan mode. During acquisition, preparations were continuously perfused *ex vivo* with room-temperature Tyrode III solution supplemented with 10 μM blebbistatin to suppress contractile motion. For pharmacological experiments, ivabradine (30 μM; Tocris), thapsigargin (1 μM; Tocris), or RU360 (5 μM; Sigma-Aldrich) was added directly to the perfusate, and preparations were incubated for 30 min before recording line scans.

During analysis, cyto-iATP and mito-iATP background-subtracted fluorescence values (F) were normalized to the baseline (F_0_) values. In a subset of experiments, cyto-iATP fluorescence signals were converted to concentration units using the “F_max_” equation (Maravall et al., 2000):

$$\left[ATP\right]=Kd\left(F⁄\left(Fmax-1⁄Rf\right)\right)/\left(1-F⁄Fmax\right),$$

where F is fluorescence, F_max_ is the fluorescence intensity of the iATP sensor in the presence of a saturating ATP concentration, K_d_ is the apparent dissociation constant of the fluorescence indicator used, and R_f_ is the indicator’s dynamic range. In the present study, F_max_ was determined under nodal imaging conditions by superfusing β-escin–permeabilized SA node preparations with 10 mM ATP, as previously described (Rhana et al., 2024). The K_d_ (1,460 μM) and R_f_ (3.8) values used were empirically determined in ventricular myocytes (Rhana et al., 2024).

For motion artifact controls, intact SA node preparations expressing the mito-iATP-HaloTag sensor were labeled by incubating the tissues for 10 min with 0.1 µM JFX554 HaloTag ligand (#HT1030; Promega). We acquired simultaneous mito-iATP and JFX554-HaloTag line-scan images and extracted signals from fixed regions of interest (ROIs) to generate baseline-normalized traces (F/F_0_, baseline = 1.0) for each channel. The ratiometric signal was then calculated as R/R_0_ = (F_mito-iATP_/F_mito-iATP,0_)/(F_HaloTag_/F_HaloTag,0_). To validate the sensitivity of the HaloTag reference channel to axial displacement under intact-node imaging conditions, controlled z-steps were imposed during two-photon acquisition by shifting the focal plane by Δz = 5 µm for 0.5 s before returning to the baseline focus. Defocus-induced excursions were quantified as the peak absolute deviation from baseline during this z-step interval.

FAD autofluorescence was imaged in SA nodes continuously perfused with warmed (35–37°C), blebbistatin-supplemented Tyrode solution. Tissues were excited at 910 nm, and emission was collected using a 570-nm dichroic mirror and a 495- to 540-nm band-pass filter. Z-stacks (2-µm step size) were acquired from the superior and inferior regions. Analysis was performed on maximum-intensity Z-projections by quantifying mean FAD intensity within defined ROIs after background subtraction.

### Electrical field stimulation and metabolic/voltage imaging of the isolated SA node

In some experiments, electrically evoked activity was induced in isolated SA node preparations via field stimulation using two platinum wire electrodes positioned on opposite sides of the tissue within the perfusion chamber. SA nodes were prepared as described above and continuously superfused with Tyrode solution containing 10 µM blebbistatin to limit motion. Baseline cytosolic ATP (cyto-iATP) and membrane voltage (ASAP5) fluorescence dynamics were first recorded during spontaneous sinus rhythm. Voltage pulses (6-ms duration) were then delivered using a Grass stimulator (AstroMed Inc.), applying the minimum voltage necessary to entrain the tissue at a frequency above the intrinsic sinus rhythm. To assess the effects of metabolic stress, the mitochondrial uncoupler FCCP was subsequently added to the perfusate. During FCCP exposure, the stimulation intensity was incrementally increased to probe electrical demand, with each voltage step maintained only for the duration required to capture stable line-scan recordings.

### Isolation of SA node myocytes

The intact SA node was excised from the beating heart and pinned flat. Single SA node myocytes were isolated using a protocol adapted from Fenske et al. (2020) and Grainger et al. (2021). Tissues were first incubated for 5 min at 37°C in a low-Ca^2+^ Tyrode solution containing 140.0 mM NaCl, 5.4 mM KCl, 0.5 mM MgCl_2_, 0.2 mM CaCl_2_, 5.0 mM HEPES, 5.5 mM glucose, 1.2 mM KH_2_PO_4_, and 50.0 mM taurine (pH 6.9, adjusted with NaOH). Enzymatic digestion was then performed for 25 min at 37°C, with gentle agitation every 5 min, using a nominally Ca^2+^-free Tyrode buffer (pH 6.9) supplemented with 18.87 U/ml elastase, 1.79 U/ml protease, 0.54 U/ml collagenase B, and 100 mg/ml bovine serum albumin (BSA).

Following digestion, tissues were rinsed twice in fresh low-Ca^2+^ Tyrode solution and twice in ice-cold Kraft–Brühe (KB) solution containing 100.0 mM L-glutamic acid (K^+^ salt), 5.0 mM HEPES, 20.0 mM glucose, 25.0 mM KCl, 10.0 mM L-aspartic acid (K^+^ salt), 2.0 mM MgSO_4_, 10.0 mM KH_2_PO_4_, 20.0 mM taurine, 5.0 mM creatine, 10.0 mM EGTA, and 1.0 mg/ml BSA (pH 7.4, adjusted with KOH). Preparations were stored at 4°C in 350 μl of KB solution for 2–3 h. Prior to mechanical dissociation, tissues were warmed to 37°C for 10 min and gently triturated using a fire-polished glass pipette. Calcium was gradually reintroduced at room temperature. First, 10 μl of a Na^+^/Ca^2+^ solution (10.0 mM NaCl and 1.8 mM CaCl_2_) was added to the cell suspension and incubated for 5 min, followed by the addition of another 23 μl of the Na^+^/Ca^2+^ solution for an additional 5 min. Finally, a BSA-supplemented Tyrode solution (140.0 mM NaCl, 5.4 mM KCl, 1.2 mM KH_2_PO_4_, 1.0 mM MgCl_2_, 1.8 mM CaCl_2_, 5.0 mM HEPES, 5.5 mM glucose, and 1.0 mg/ml BSA) was added in three sequential steps of 55, 175, and 612 µl at 4-min intervals.

### Confocal imaging of [Ca^2+^]_i_, [ATP]_i_, [ATP]_mito_, and mitochondria in isolated SA node myocytes

Intracellular Ca^2+^ and cyto-iATP or mito-iATP signals from dissociated SA node myocytes were simultaneously recorded using our inverted Olympus FluoView FV3000 confocal microscope operating in 2D or line-scan mode. To image cytosolic Ca^2+^ signals, SA node myocytes expressing cyto-iATP or mito-iATP were loaded with the red-shifted Ca^2+^ indicator Rhod-3 AM (3 µM; Thermo Fisher Scientific), as described previously (Rhana et al., 2024). After loading cells with Rhod-3, a drop of the cell suspension was transferred to a temperature-controlled (37°C) perfusion chamber on a microscope stage, and cells were allowed to settle onto the coverslip for 5 min. Fluorescent sensors were excited with solid-state lasers emitting at 488 nm (cyto-iATP and mito-iATP) or 561 nm (Rhod-3) through a 60× oil-immersion lens (PlanApo; NA = 1.40). During analysis, background was subtracted from all confocal images, and fluorescence signals were expressed as F/F_0_, where F is the fluorescence intensity at a given time point, and F_0_ is the mean baseline fluorescence. Ca^2+^, cyto-iATP, and mito-iATP signals were automatically detected and analyzed using ImageJ and SanPy software (Guarina et al., 2024).

In some experiments, Ca^2+^ release events were recorded from SA node myocytes loaded with Fluo-4 AM. These signals were detected and analyzed using an ML-based Ca^2+^ signal detection and analysis pipeline as previously described (Garrud et al., 2024). In brief, a F/F_0_ channel was calculated from the raw 488 channel by dividing each frame by a five-frame moving average (without background subtraction) and rescaled to eliminate fractional pixel values. Both channels (488 and F/F_0_) were used to simultaneously train a ML model ground truth by manually annotating every pixel of at least 10 Ca^2+^ signals from 10 representative experiments (Arivis Vision4D, Zeiss). The trained ML model was run on all experiments, and Ca^2+^ signals with a mean F/F_0_ intensity of <1.025 of baseline or a total area of <5 μm were excluded from further analysis. ML-annotated Ca^2+^ signal properties (size, location [x, y, t], duration, and signal mass [sum of each Ca^2+^ signal F/F_0_ over its duration]) were exported for summary and statistical analysis.

For isolated-cell motion artifact controls, dissociated SA node myocytes expressing mito-iATP-HaloTag were labeled by incubating the cells for 10 min with 0.1 µM JFX554 HaloTag ligand. Cells were then transferred to a recording chamber containing Tyrode’s solution (37°C) and imaged using 488-nm (mito-iATP) and 561-nm (HaloTag-JFX554) excitation. Simultaneous line-scan images of mito-iATP and JFX554 were acquired, and signals were extracted from fixed ROIs to generate baseline-normalized traces (F/F_0_, baseline = 1.0) for each channel. The ratiometric signal (R/R_0_) was then calculated exactly as described above for intact-node preparations.

### Super-resolution imaging of SA node myocytes

For these experiments, isolated SA node myocytes expressing cyto-iATP or mito-iATP were incubated with 250 nM MitoTracker Deep Red (Thermo Fisher Scientific) for 30 min at 37°C. Super-resolution radial fluctuation (SRRF) imaging was performed using a Dragonfly 200 spinning-disk confocal system (Andor Technology) equipped with an Andor iXon EMCCD camera. This system was coupled to an inverted Leica DMi microscope equipped with a 60× oil-immersion objective with a NA of 1.4. Images were acquired using Fusion software (Andor, UK). Colocalization of ATP sensors with MitoTracker was evaluated using the colocalization module in Imaris 10 (Oxford Instruments).

### Quantification of mitochondrial volume

Mitochondrial volume was quantified by analyzing SRRF-acquired 3D image stacks of MitoTracker-labeled SA node myocytes with Imaris 10 software (Oxford Instruments). Myocytes were isolated from anatomically defined superior and inferior regions of the SA node. Mitochondria were segmented using the Surfaces tool based on MitoTracker Deep Red fluorescence intensity, applying a consistent threshold across all samples. Total mitochondrial volume was computed for each individual cell, allowing for regional comparisons between superior and inferior SA node myocyte populations.

### SA node cryosectioning and RNAscope *in situ* hybridization

The SA node region was fixed in 4% paraformaldehyde for 30 min, washed in PBS (0.01 M), and dehydrated in PBS containing 30% sucrose for 24 h prior to embedding in OCT medium (Sakura, USA). Tissues were oriented perpendicular to the long axis of the SA node during embedding to allow for longitudinal sectioning. Cryosections (5-μm thickness) were cut and mounted onto glass slides. *In situ* hybridization was then performed on these sections using RNAscope 2.5 HD Duplex Assay Kit (Advanced Cell Diagnostics) with the Mm-ATP5A1-C2 probe (#459311-C2) and hematoxylin counterstaining, following the manufacturer’s standard protocol. Hematoxylin counterstaining was used for anatomical reference and confirmation of SA node regional identity. ATP5A1-positive puncta were visualized using Fast Red chromogenic detection, and their fluorescence was imaged on an APX100 microscope equipped with a UPLXAPO 40× objective (NA 0.95; Olympus) using a TRITC filter cube. A single projected image resolving puncta throughout the tissue thickness was generated from Z-stacks spanning the full section thickness (5 μm, 14 optical planes), acquired using the extended focal imaging and stitching functions in CellSens APEX 4.3 software. Image analysis was performed using ImageJ version 2.14.0 (NIH). Rectangular ROIs (250 × 25 μm) were positioned along the long axis of the SA node. ROIs were preprocessed using a 2-pixel-radius median filter followed by rolling-ball background subtraction (radius, 30 pixels). Local fluorescence maxima, corresponding to both isolated and clustered ATP5A1 RNA puncta, were identified using the Find Maxima function with a fixed prominence threshold. The total number of detected maxima within each ROI was normalized to the ROI area and reported as punctate density (puncta per mm^2^).

### Statistics

Data were analyzed using hierarchical statistics to account for the nested structure of the data (cells within animals). Per-cell values were first summarized within each animal, and animals were treated as the unit of inference (*N* in figures represents the number of animals). Where variance differed between groups (heteroskedasticity assessed by Brown–Forsythe), Welch’s ANOVA or Welch’s *t* test with Games–Howell post-hoc comparisons was used. Distributions were analyzed using finite Gaussian-mixture models, with component selection based on the Bayesian information criterion. Statistical significance was set at P < 0.05; individual P values are denoted in figures. Distributions that were statistically multimodal (i.e., ATP signal mass, Ca^2+^ signal mass, and mitochondrial volume fraction) were modeled with finite Gaussian-mixture models. The optimal number of components was selected by minimizing the Bayesian information criterion and confirmed by the gap statistic, silhouette score, and Hartigan’s dip test for unimodality (P < 0.001 for departure from a single mode).

### Online supplemental material

**Figure S1.**
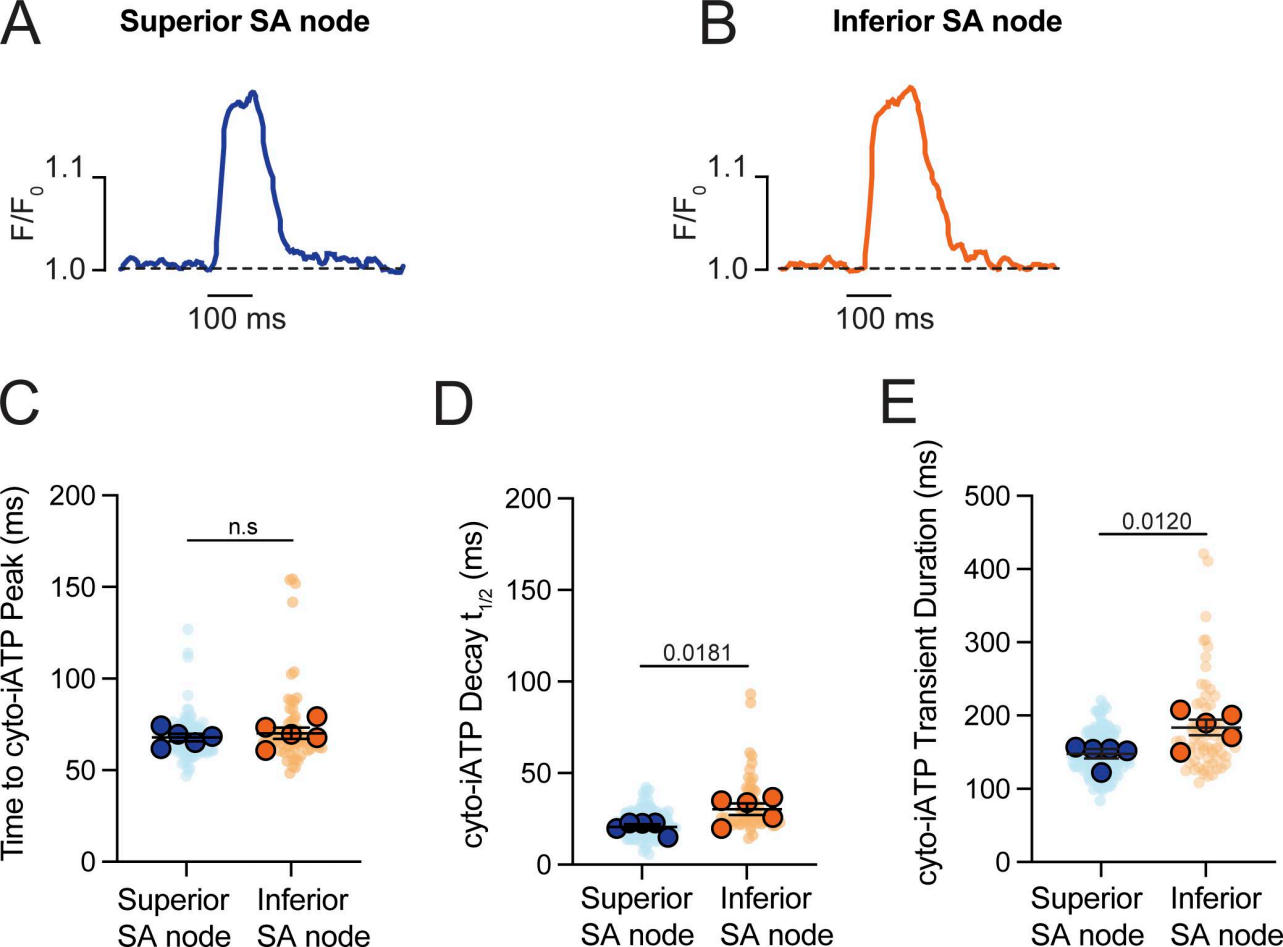
**Accelerated ATP decay and shorter transient duration characterize the high-flux phenotype of the superior SA node. (A and B)** Representative time course traces of cyto-iATP transients recorded from superior (A) and inferior (B) SA node regions. Traces are normalized to peak amplitude to facilitate comparison of transient kinetics. The dashed line indicates baseline fluorescence (F/F_0_ = 1). **(C–E)** Summary quantification of cyto-iATP kinetic parameters (*N* = 5 mice per group), including time to peak (C), decay t_1/2_ (D), and total transient duration (E). P values are indicated in the text (time to peak, P = 0.2777; decay t_1/2_, P = 0.0181; duration, P = 0.0120). Large circles denote per-animal means; small circles indicate individual biological replicates. *N* represents the number of independent mice.

**Figure S2.**
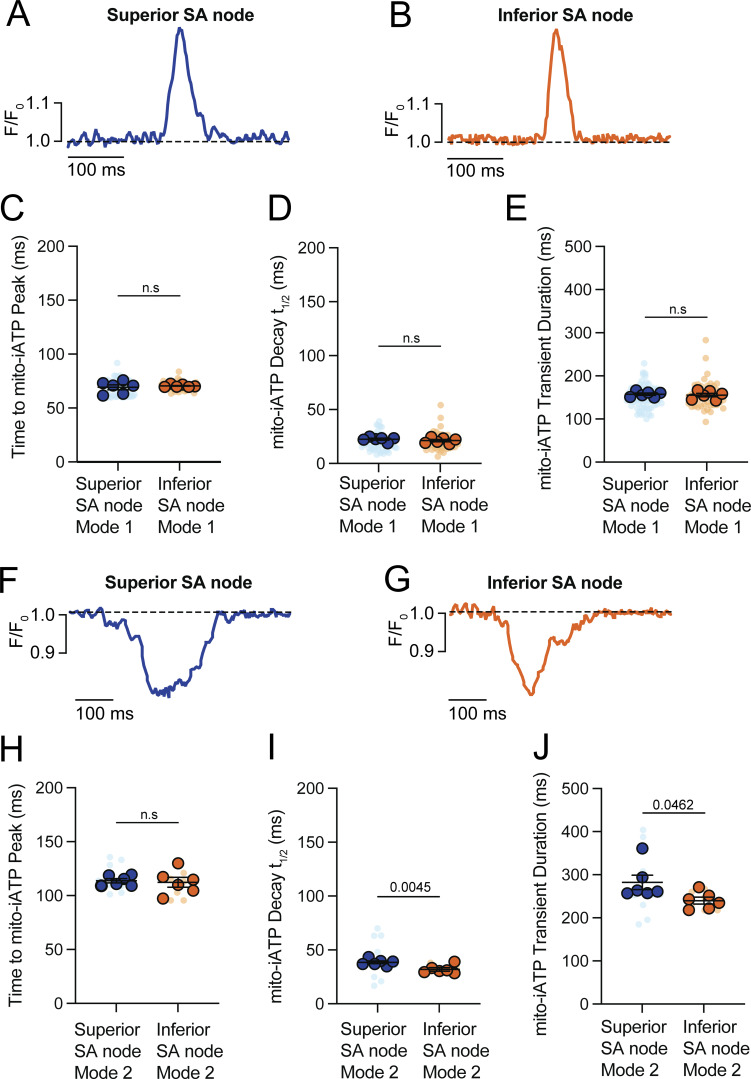
**Mitochondrial ATP synthesis kinetics is regionally conserved, whereas consumption duration scales with metabolic load. (A–E)** Kinetic analysis of Mode 1 (ATP production) mito-iATP transients. **(A and B)** Representative time course traces from superior (A) and inferior (B) SA node regions. Traces are normalized to peak amplitude to facilitate comparison of temporal kinetics. The dashed line indicates baseline fluorescence (F/F_0_ = 1). **(C–E)** Summary quantification of time to peak (C), decay t_1/2_ (D), and transient duration (E) (*N* = 6 mice per group). **(F–J)** Kinetic analysis of Mode 2 (ATP consumption) mito-iATP transients. **(F and G)** Representative time course traces from superior (F) and inferior (G) SA node regions showing negative mito-iATP deflections associated with ATP consumption. **(H–J)** Summary quantification of time to peak (H), decay t_1/2_ (I), and transient duration (J). P values are shown above comparisons. Large circles denote per-animal means; small circles indicate individual biological replicates. *N* represents the number of independent mice.

**Figure S3.**
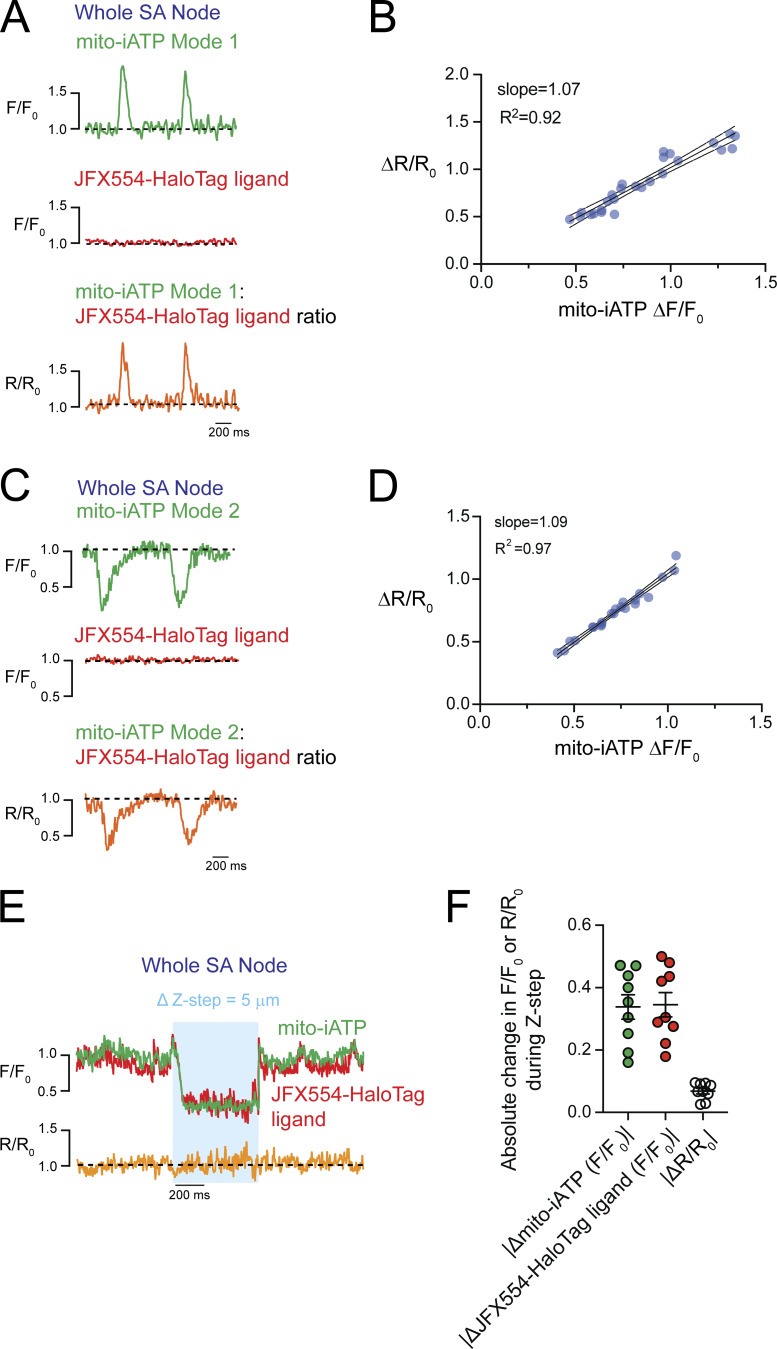
**Baseline-normalized ratiometry rules out motion and focus artifacts, confirming that beat-locked mito-iATP oscillations are genuine metabolic signals. (A and C)** Representative recordings from intact SA node preparations exhibiting Mode 1 (A) and Mode 2 (C) mito-iATP dynamics. The ATP sensor channel (mito-iATP, green) is shown together with a spectrally distinct reference channel (JFX554–HaloTag, red); the baseline-normalized ratiometric trace (R/R_0_, orange) is overlaid. Traces are shown as normalized fluorescence (F/F_0_; baseline = 1.0; dashed line). Data in A–D were obtained from three independent SA node preparations (*N* = 3); 26 events are shown in B and 22 events in D. **(B and D)** Event-level relationship between mito-iATP peak amplitude (ΔF/F_0_) and ratiometric peak amplitude (ΔR/R_0_) for Mode 1 (B) and Mode 2 (D) events. Each symbol represents one mito-iATP transient event. Solid lines indicate linear fits to Mode 1 (slope = 1.07, R^2^ = 0.92) and Mode 2 (slope = 1.09, R^2^ = 0.97) events, with shaded regions denoting 95% confidence intervals. Signals were extracted from fixed ROIs and analyzed as F/F_0_ (baseline = 1.0). The ratiometric signal was computed as (R/R_0_ = (F_mito-iATP_/F_mito-iATP,0_)/(F_HaloTag_/F_HaloTag,0_)). **(E)** Representative recording during controlled axial displacement (Δz-step = 5 µm) in an intact SA node preparation. The shaded region indicates the imposed z-step interval. Mito-iATP (green) and JFX554–HaloTag (red) are shown as F/F_0_ (baseline = 1.0), with (R/R_0_) overlaid (orange). **(F)** Within this representative node, peak absolute deviation from baseline during the z-step interval is summarized for mito-iATP (|ΔF/F_0_|), JFX554–HaloTag (|ΔF/F_0_|), and the ratiometric signal (|Δ(R/R_0_)|). Each symbol represents one ROI/cell measured within the same preparation (*N* = 1 SA node; *n* = 9 ROIs/cells). *N* represents the number of independent mice.

**Figure S4.**
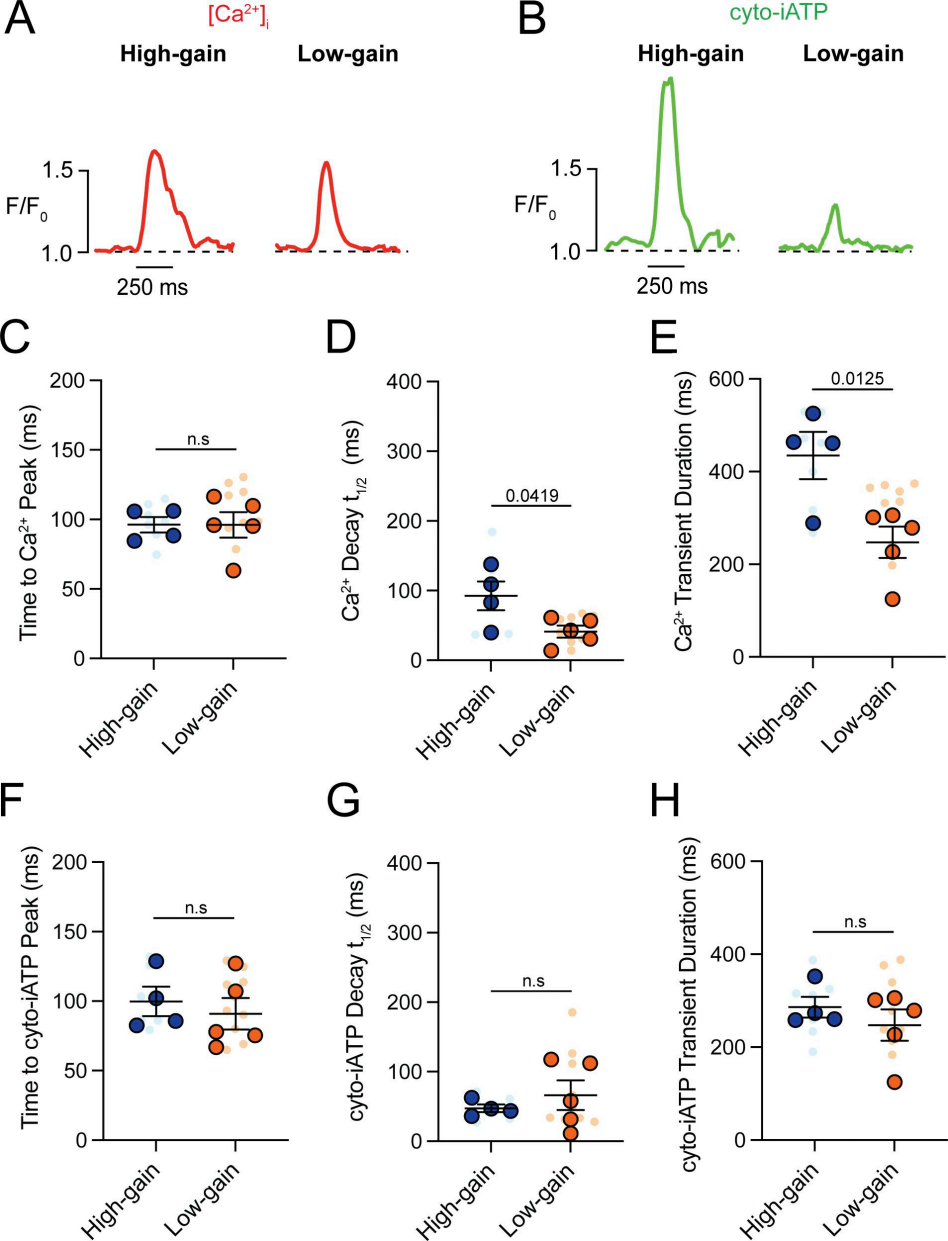
**High-gain SA node myocytes exhibit prolonged intracellular Ca** ^**2+**^ **transients compared with low-gain myocytes. (A and B)** Representative normalized confocal line-scan recordings of intracellular Ca^2+^ (red, A) and cyto-iATP (green, B) from myocytes classified as high-gain (left) or low-gain (right). The dashed line indicates baseline fluorescence (F/F_0_ = 1). **(C–E)** Summary quantification of Ca^2+^ transient kinetics (high-gain, *N* = 4; low-gain, *N* = 5), including time to peak (C), decay t_1/2_ (D), and transient duration (E). **(F–H)** Summary quantification of cyto-iATP transient kinetics, including time to peak (F), decay constant (G), and transient duration (H). P values are shown above comparisons. Large circles denote per-animal means; small circles indicate individual biological replicates. *N* represents the number of independent mice.

**Figure S5.**
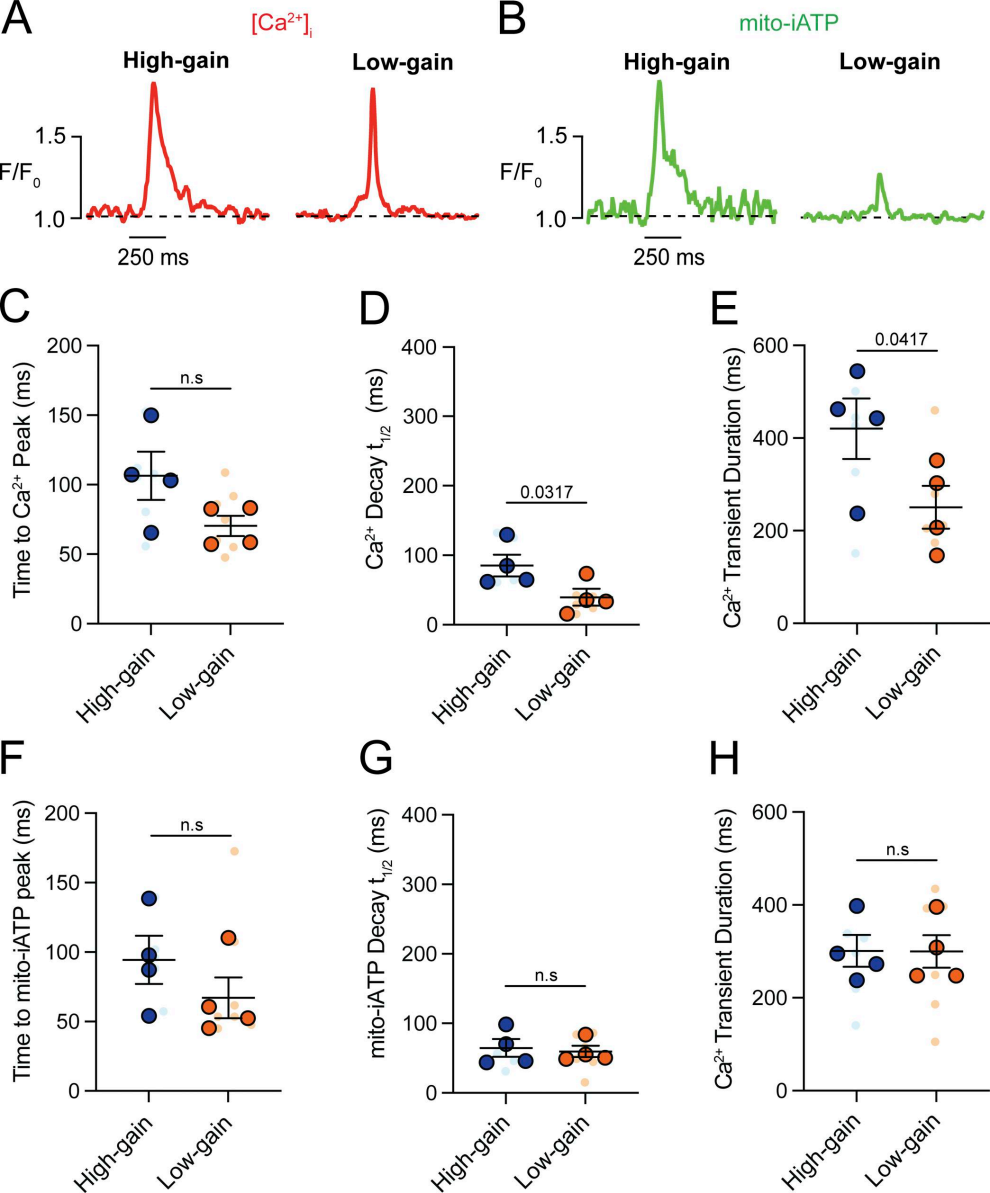
**High-gain mitochondrial ATP synthesis is associated with prolonged intracellular Ca** ^**2+**^ **transients. (A and B)** Representative normalized confocal line-scan recordings of intracellular Ca^2+^ (red, A) and mito-iATP (green, B) from myocytes classified as high-gain (left) or low-gain (right). The dashed line indicates baseline fluorescence (F/F_0_ = 1). **(C–E)** Summary quantification of Ca^2+^ transient kinetics (*N* = 4 mice per group), including time to peak (C), decay t_1/2_ (D), and transient duration (E). **(F–H)** Summary quantification of Mode 1 mito-iATP transient kinetics, including time to peak (F), decay t_1/2_ (G), and transient duration (H). P values are shown above comparisons. Large circles denote per-animal means; small circles indicate individual biological replicates. *N* represents the number of independent mice.

**Figure S6.**
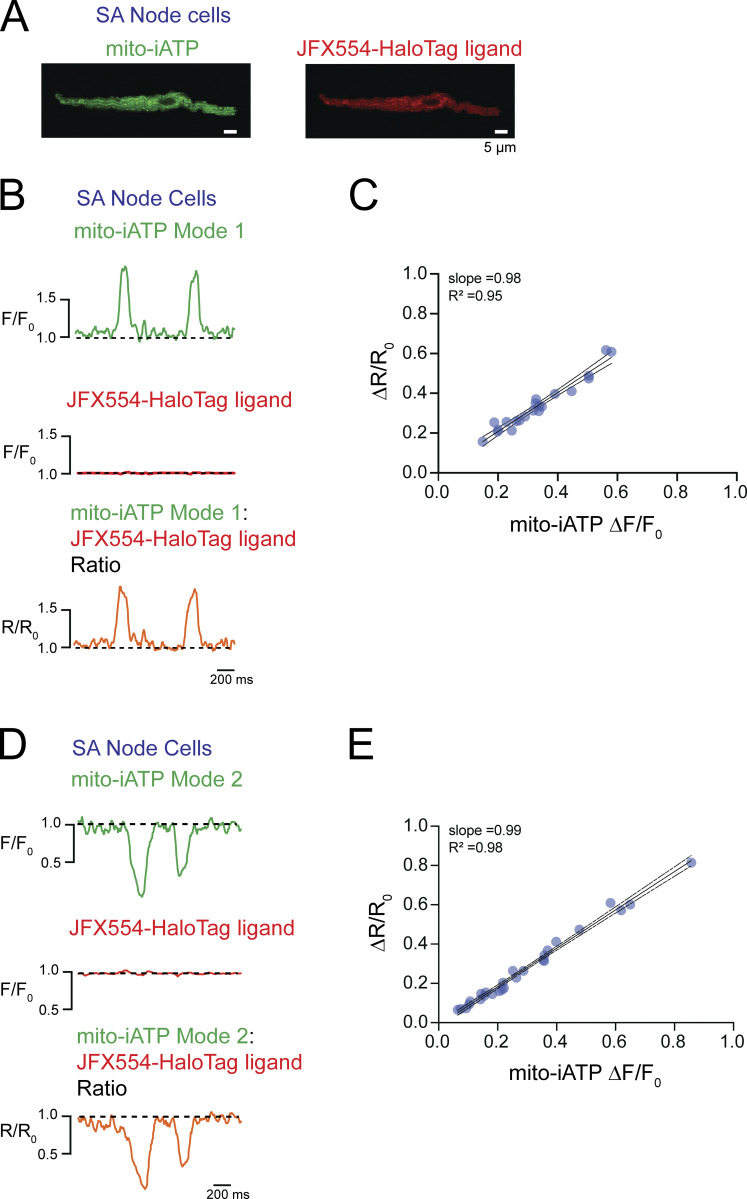
**Ratiometric validation of mito-iATP signals in isolated SA node myocytes rules out optical artifacts, confirming that Mode 1 and Mode 2 transients reflect genuine metabolic signals. (A)** Representative fluorescence image of an isolated SA node myocyte expressing mito-iATP (green) and labeled with the reference fluorophore JFX554-HaloTag ligand (red). Scale bar, 5 µm. **(B and D)** Representative single-cell recordings showing Mode 1 mito-iATP transients (B) and Mode 2 mito-iATP transients (D). The baseline-normalized mito-iATP signal (F/F_0_; green) is shown alongside the reference fluorophore signal (JFX554-HaloTag; red) and the resulting ratiometric trace (R/R_0_; orange). Dashed lines denote baseline fluorescence. Scale bars: 200 ms. **(C and E)** Event-level relationship between the mito-iATP peak amplitude (F/F_0_) and the ratiometric amplitude (R/R_0_) for Mode 1 (C) and Mode 2 (E) events. Each blue circle represents an individual transient. Solid lines indicate linear fits (Mode 1: slope = 0.98, R^2^ = 0.95; Mode 2: slope = 0.99, R^2^ = 0.98), with shaded regions representing 95% confidence intervals. Data in A–E were obtained from SA node cells isolated from three mice (*N* = 3); C shows 21 events, and E shows 27 events. *N* represents the number of independent mice.

**Figure S7.**
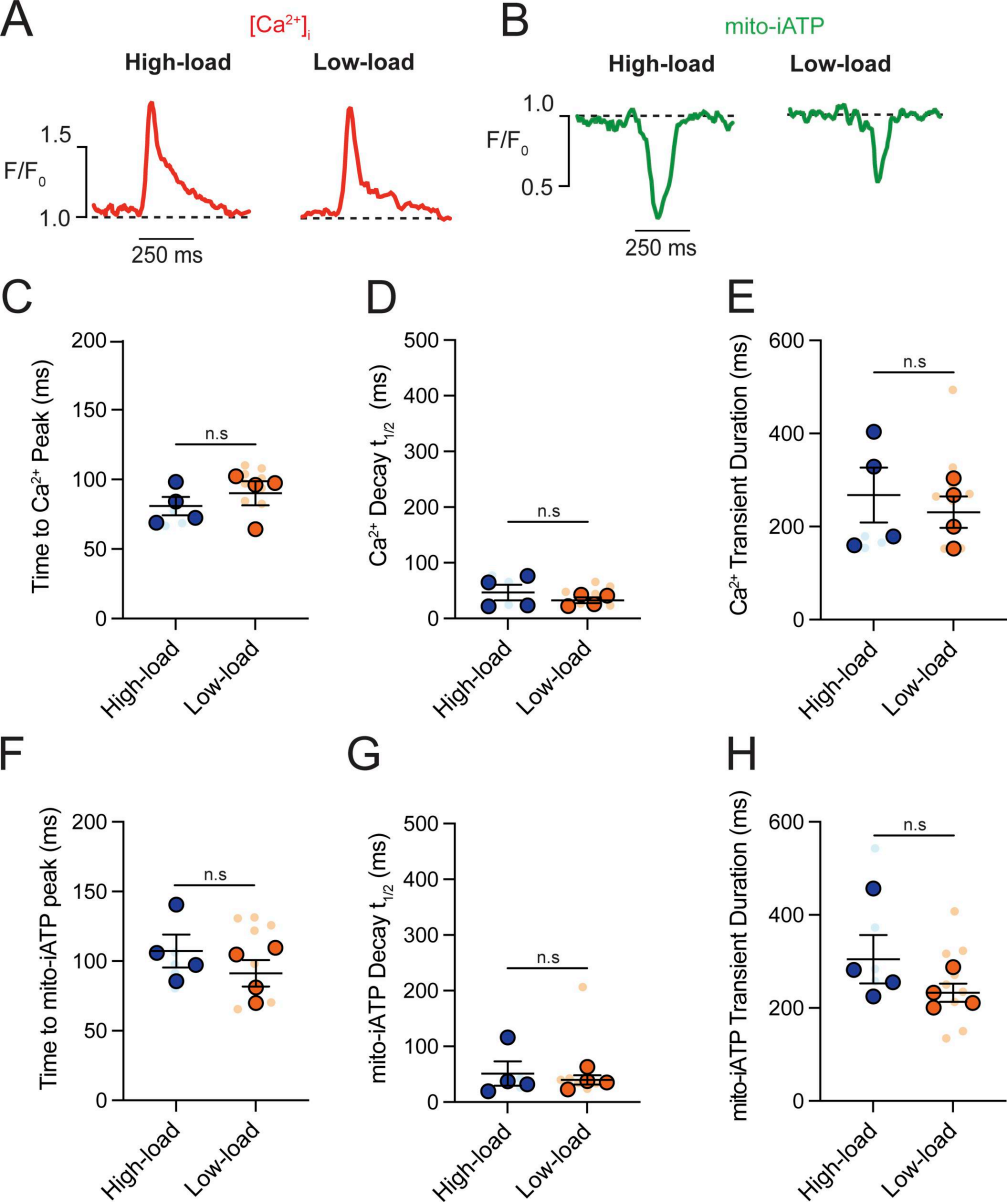
**High-load mitochondrial ATP consumption is associated with intracellular Ca** ^**2+**^ **release kinetics in SA node myocytes. (A and B)** Representative normalized confocal line-scan recordings of intracellular Ca^2+^ (red, A) and Mode 2 mito-iATP signals (green, B) from myocytes classified as high-load (left) or low-load (right). The dashed line indicates baseline fluorescence (F/F_0_ = 1). **(C–E)** Summary quantification of Ca^2+^ transient kinetics (*N* = 4 mice per group), including time to peak (C), decay t_1/2_ (D), and transient duration (E). **(F–H)** Summary quantification of Mode 2 mito-iATP transient kinetics. P values are shown above comparisons. Large circles denote per-animal means; small circles indicate individual biological replicates. *N* represents the number of independent mice.

Figs. S1 and S2 quantify the kinetics of beat-locked ATP signals in the intact SA node, with Fig. S1 reporting the rise/decay parameters of whole-node cyto-iATP transients and Fig. S2 reporting the corresponding kinetic analysis of whole-node mito-iATP Mode 1 and Mode 2 events. Fig. S3 provides ratiometric controls using ATP-insensitive HaloTag ligand JFX554 to validate that beat-locked mito-iATP oscillations are not driven by motion or optical-path fluctuations in intact nodes. Figs. S4 and S5 focus on acutely dissociated SA node myocytes and relate intracellular Ca^2+^ handling to ATP dynamics at the single-cell level. Fig. S6 provides ratiometric controls using ATP-insensitive HaloTag ligand JFX554 to validate that beat-locked mito-iATP oscillations are not driven by motion or optical-path fluctuations in isolated myocytes. Fig. S7 shows the kinetic analysis of Ca^2+^ and mito-iATP signals in high- and low-load sites.

## Results

### Superior and inferior regions of the SA node exhibit distinct cytosolic ATP dynamics

**Figure 1.**
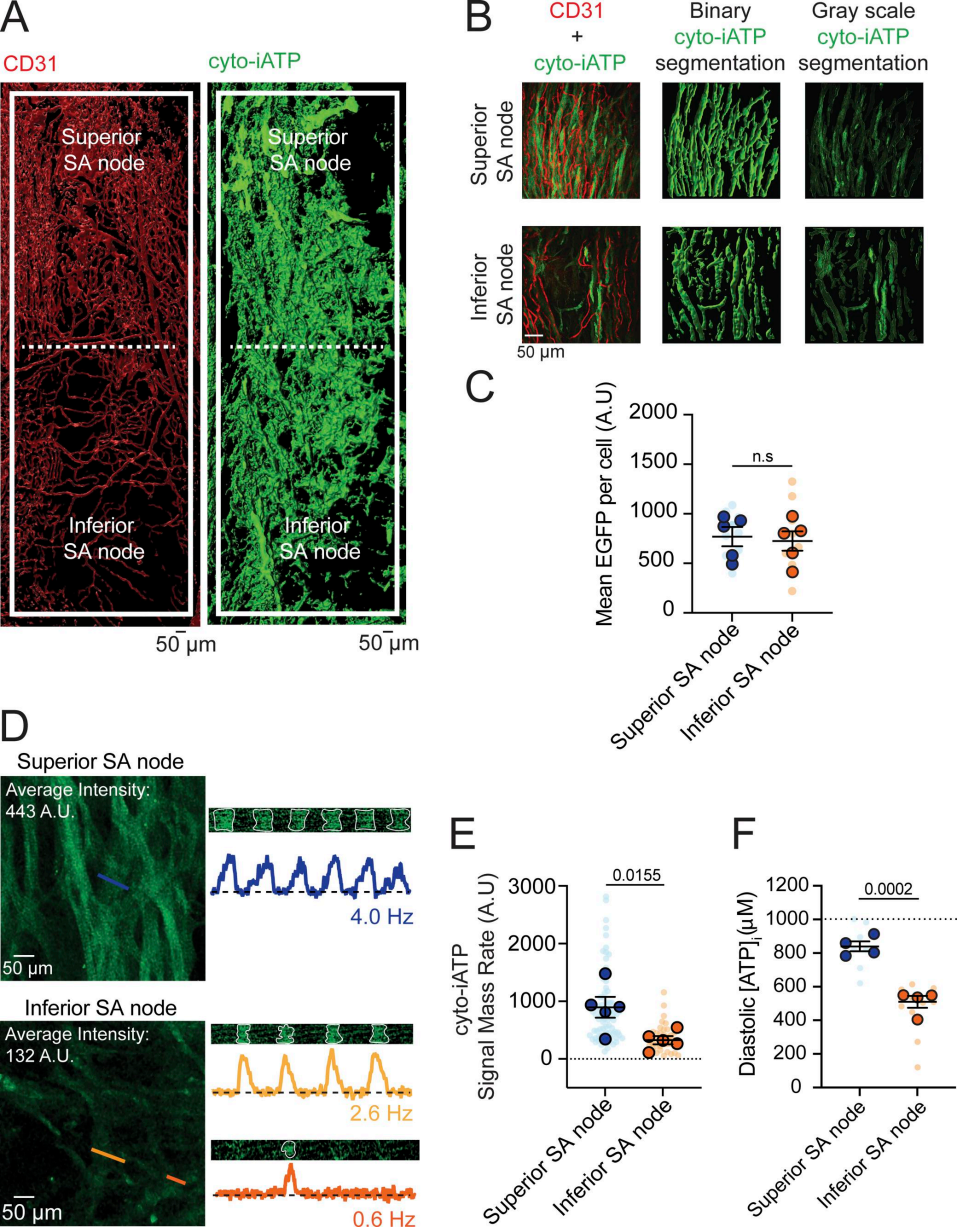
**Superior SA node myocytes exhibit elevated diastolic ATP and metabolic flux compared with the inferior region. (A)** 3D segmented maximum-intensity projection of a whole-mount SA node immunolabeled for CD31 (vasculature, red) and cyto-iATP (myocytes, green). The dashed line denotes the boundary between superior and inferior regions. **(B)** Image-processing workflow illustrating merged maximum-intensity projections, binary segmentation masks, and extraction of grayscale cyto-iATP signals used for quantitative analysis. **(C)** Mean cyto-iATP fluorescence intensity per myocyte, grouped by region (*N* = 5 mice per region), reporting expression levels of the EGFP-tagged cyto-iATP sensor. **(D)** Live confocal imaging of cyto-iATP signals showing representative line-scan images and corresponding normalized fluorescence traces (F/F_0_) from superior and inferior regions. **(E and F)** Summary quantification of cyto-iATP signal mass rate (E) and estimated diastolic [ATP]_i_ (F). P values are shown above comparisons. Large circles denote per-animal means; small circles indicate individual biological replicates. *N* represents the number of independent mice.

To map regional ATP dynamics, we first expressed the genetically encoded cytosolic ATP reporter, iATPSnFR1 (cyto-iATP) (Lobas et al., 2019), in mouse SA node myocytes, then surveyed cyto-iATP–associated fluorescence throughout intact mouse SA node whole mounts (Fig. 1). Preparations were costained with the endothelial marker, CD31, to provide vascular landmarks and demarcate SA node borders. In agreement with prior work (Grainger et al., 2021; Manning et al., 2025), vessel density varied markedly along the mouse node and was highest in the superior region (Fig. 1 A). Cyto-iATP signals were detected throughout the node, and cyto-iATP fluorescence was diffusely distributed in isolated SA node myocytes. Automated segmentation revealed more cyto-iATP–positive myocytes in the superior than the inferior region, consistent with the previously reported SA node myocyte density gradient; however, mean cyto-iATP fluorescence per cell did not differ between superior and inferior regions (Fig. 1, B and C).

We next imaged cyto-iATP *ex vivo* in intact SA nodes during sinus rhythm using multiphoton microscopy while suppressing contraction with blebbistatin (10 µM) to eliminate motion artifacts. In these live preparations, optical sections consistently exhibited higher regional mean cyto-iATP fluorescence in the superior domain than in the inferior domain (Fig. 1 D). Because mean per-cell EGFP-tagged cyto-iATP fluorescence did not differ in the whole-mount analysis (Fig. 1 C), the elevated regional signal in live tissue is consistent with the hypothesis that steady-state cytosolic ATP availability is higher in the superior pacemaker zone.

Line-scan multiphoton imaging further revealed large, rhythmic cyto-iATP transients in the superior SA node that tracked the local firing rate (∼4 Hz; Fig. 1 D, top). Inferior regions were more heterogeneous: some myocytes fired more slowly (∼2.6 Hz), whereas others were nearly quiescent (∼0.6 Hz; Fig. 1 D, middle and bottom). Although action potentials were not recorded in these experiments, the observed frequencies closely match reported electrical measurements in superior and inferior SA node myocytes, supporting the interpretation that cyto-iATP oscillations are beat-locked readouts of intrinsic pacemaker activity (Grainger et al., 2021).

A kinetic analysis showed similar time to peak for superior (67.85 ± 2.11 ms) and inferior (70.14 ± 3.05 ms) cyto-iATP transients (P = 0.2777; Fig. S1). In contrast, decay phases, measured as time to 50% amplitude (t_1/2_), were slower in inferior cells (t_1/2_ = 30.25 ± 3.22 ms) than superior cells (t_1/2_ = 20.55 ± 1.52 ms; P = 0.0181), yielding a longer overall transient duration in the inferior region (183.70 ± 10.53 ms) compared with the superior region (147.80 ± 6.41 ms; P = 0.0120).

To quantify beat-to-beat energetic output independent of absolute calibration, we computed the cyto-iATP signal mass of each transient, defined as the time- and area-integrated change in fluorescence. A compilation of all individual transients over identical recording durations (4.2 s) showed that the total cyto-iATP signal mass was markedly greater in cells of the superior SA node (895.70 ± 180.80 AU) than in inferior cells (326.40 ± 71.75 AU). Consistently, across nodes, the superior region exhibited a higher signal mass rate (total signal mass per trace divided by recording time; Fig. 1 E; P = 0.0155). Thus, superior SA node myocytes sustain higher beat-to-beat energetic throughput than inferior myocytes.

To estimate regional differences in absolute ATP concentration, we converted cyto-iATP fluorescence to [ATP]_i_ using an F_max_-based approach (Maravall et al., 2000). F_max_ was determined by permeabilizing SA nodes expressing cyto-iATP with β-escin followed by exposure to saturating (10 mM) ATP; [ATP]_i_ was then computed by combining F_max_ with prior *in situ* sensor parameters (Rhana et al., 2024). Using this approach, we found that diastolic [ATP]_i_ was sub-millimolar in both regions but was significantly higher in the superior SA node (839.9 ± 29 µM) than in the inferior SA node (510.1 ± 35.24 µM; P = 0.0002), consistent with the higher calibration-independent cyto-iATP signal mass rate in superior cells (Fig. 1 F).

### Pharmacological dissection of I_f_ and SR Ca^2+^ contributions to cytosolic ATP dynamics

**Figure 2.**
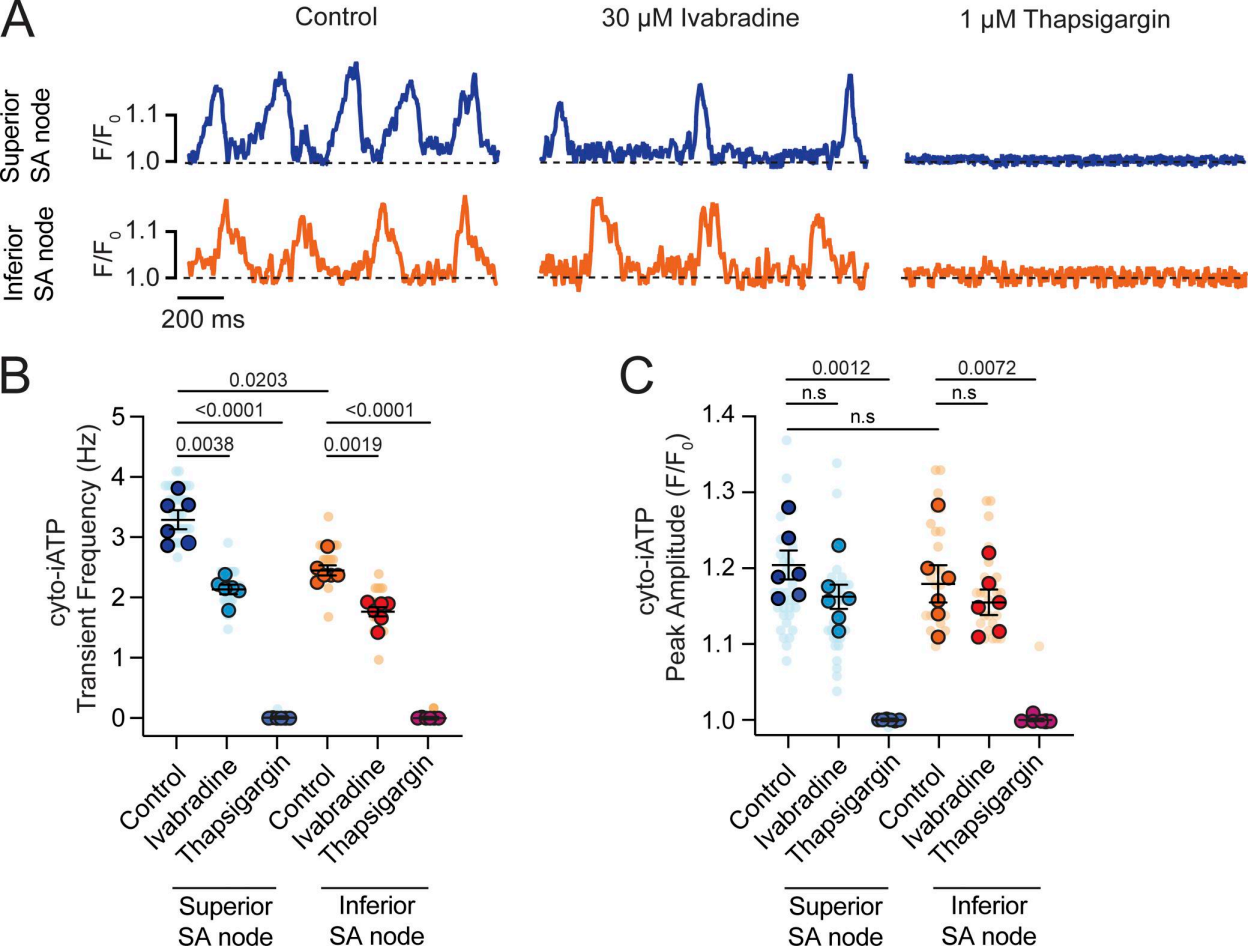
**SR Ca** ^**2+**^ **release drives beat-to-beat ATP oscillations, while I**_**f**_ **modulates metabolic frequency. (A)** Representative normalized cyto-iATP confocal line-scan traces from superior (top) and inferior (bottom) SA nodes acquired under control conditions (left), after I_f_ inhibition with ivabradine (30 µM; middle), and following SERCA inhibition with thapsigargin (1 µM; right). Traces are shown as normalized fluorescence (F/F_0_). The dashed line indicates baseline fluorescence (F/F_0_ = 1). **(B and C)** Summary quantification of cyto-iATP oscillation frequency (B) and peak cyto-iATP oscillation amplitude (C) across conditions (*N* = 6 mice per group). P values are shown above comparisons. Large circles denote per-animal means; small circles indicate individual biological replicates. *N* represents the number of independent mice.

We next asked how two key contributors to pacemaker cycling—I_f_-dependent depolarization and SR Ca^2+^ cycling/whole-cell Ca^2+^ transients—shape energetic demand in the SA node (Fig. 2). To address this, we imaged cyto-iATP–expressing nodes in sinus rhythm before and after inhibiting I_f_-producing HCN channels with ivabradine or suppressing SR Ca^2+^ release with the SERCA inhibitor thapsigargin (Fig. 2 A). Under basal conditions, superior SA node regions exhibited rhythmic cyto-iATP transients at 3.27 ± 0.06 Hz (*N* = 6 SA nodes), whereas inferior regions fired more slowly at 2.12 ± 0.07 Hz (*N* = 6 SA nodes; P = 0.0203), but their amplitude (1.17 ± 0.02 F/F_0_; P = 0.9978) was similar to that of superior cells (1.20 ± 0.01 F/F_0_; P = 0.7433).

Ivabradine (30 µM) lengthened the cycle, decreasing frequency from 3.27 ± 0.06 Hz to 2.12 ± 0.07 Hz in superior cells (P = 0.0038) and from 2.45 ± 0.08 Hz to 1.76 ± 0.07 Hz in inferior cells (P = 0.0019), but did not alter the transient peak (F/F_0_: 1.16 ± 0.01 and 1.15 ± 0.01 in superior and inferior SA nodes, respectively) (Fig. 2, B and C). Thus, HCN channel inhibition curtailed ATP demand chiefly by lowering beat rate. In contrast, blocking SERCA with thapsigargin (1 µM) abolished spontaneous firing, collapsing cyto-iATP transients to baseline, and prevented recovery during the 10-min recording (Fig. 2, B and C). The complete loss of ATP pulses despite an intact membrane clock indicates that SR Ca^2+^ cycling is essential not only for excitability but also for beat-to-beat ATP generation.

Together, these manipulations suggest that chronotropic control and SR Ca^2+^ reuptake make separable, complementary contributions to cellular ATP turnover. The high metabolic output characteristic of the superior pacemaker zone is critically dependent on continuous SERCA activity, underscoring the role of Ca^2+^ release as a master regulator of metabolic supply rather than a passive follower of electrical activity.

### Ca^2+^ transient–locked mitochondrial ATP transients are stronger in superior SA node myocytes

**Figure 3.**
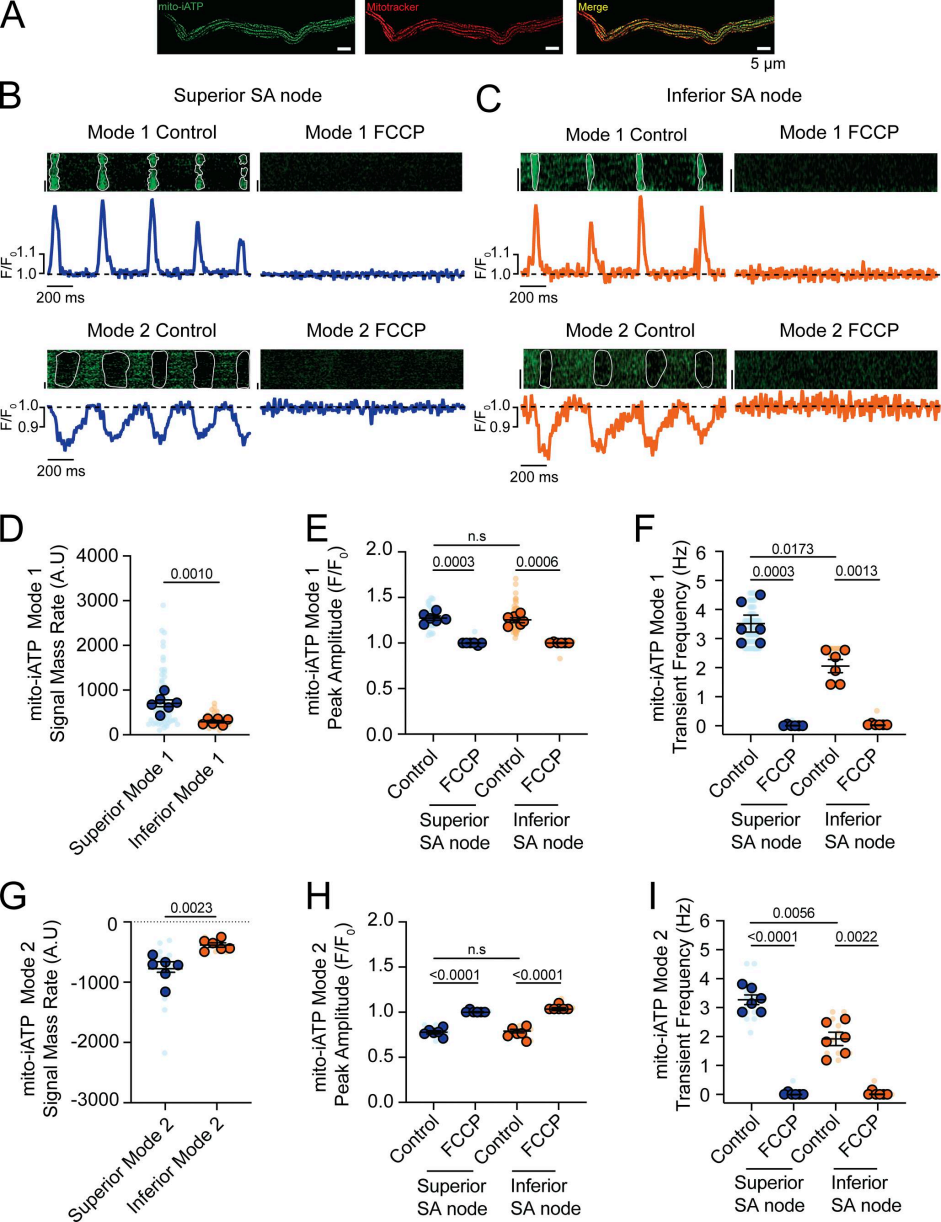
**Oxidative phosphorylation drives distinct modes of beat-to-beat ATP dynamics in SA node mitochondria. (A)** Representative confocal image of an isolated SA node myocyte co-expressing mito-iATP (green) and labeled with MitoTracker (red), illustrating mitochondrial localization of the mito-ATP sensor. Scale bar, 5 µm. **(B and C)** Representative confocal line-scan images and corresponding normalized fluorescence traces (F/F_0_) from superior (B) and inferior (C) SA node regions. The dashed line indicates baseline fluorescence (F/F_0_ = 1). Two classes of mito-iATP signals are resolved: Mode 1 transients, characterized by positive deflections, and Mode 2 transients, characterized by negative deflections. Traces acquired after application of the mitochondrial uncoupler FCCP (1 µM) are shown for comparison. **(D–F)** Summary quantification of Mode 1 mito-iATP dynamics, including signal mass rate (D), peak amplitude (E), and transient frequency (F). **(G–I)** Summary quantification of Mode 2 mito-iATP dynamics, including signal mass rate (G), peak amplitude (H), and transient frequency (I). P values are shown above comparisons. Data are presented as means ± SEM (*N* = 6 mice per group). Large circles denote per-animal means; small circles indicate individual biological replicates. *N* represents the number of independent mice.

To test whether cyto-iATP oscillations arise from beat-locked changes in mitochondrial ATP, we recorded line-scan images across an intact, mito-iATP–expressing SA node (Fig. 3). The mitochondrial reporter (mito-iATP) is derived from iATPSnFR2 (A95A/A119L low-affinity variant; apparent K_d_ ≈ 0.5 mM) and was targeted to the mitochondrial matrix using four tandem COX8 leader sequences (Marvin et al., 2024). Fig. 3 A shows an isolated SA node myocyte expressing mito-iATPSnFR2 and stained with MitoTracker. mito-iATPSnFR2 displayed the expected punctate mitochondrial pattern and strong spatial overlap with MitoTracker Far Red (mean Manders’ coefficient, 0.85 ± 0.03), consistent with robust mitochondrial targeting and in agreement with prior validation of mito-iATPSnFR2 targeting/behavior in Rho° cells and neurons (Marvin et al., 2024), as well as in cardiomyocytes (Rhana et al., 2025, *Preprint*).

Having confirmed the selective mitochondrial targeting of mito-iATP in SA node pacemaker cells, we proceeded to perform high-resolution imaging of mitochondrial ATP dynamics. Spontaneous, rhythmic mitochondrial signals were detected in both superior and inferior regions of the node, appearing as two stereotyped waveforms, designated Mode 1 and Mode 2 (Fig. 3, B and C). Mode 1 events exhibited a rapid rise after each beat, followed by decay, whereas Mode 2 events showed a transient dip before recovering, presumably as new ATP molecules were synthesized.

We performed a detailed kinetic analysis of mito-iATP events in the intact SA node (Fig. S2). For Mode 1 “gain” events, rise and decay kinetics were statistically indistinguishable in superior and inferior SA nodes; time to peak was 69.23 ± 2.29 ms in the superior region and 70.58 ± 0.52 ms in the inferior region, followed by decay with a time to 50% amplitude (t_1/2_) of 22.48 ± 0.86 ms in the superior region and 21.21 ± 1.05 ms in the inferior region (Fig. S2, A–E). Mode 2 “dip” events reached a similar nadir in the superior node (113.70 ± 1.94 ms) and the inferior node (112.30 ± 4.59 ms) (P = 0.7784); recovery proceeded with higher t_1/2_ values of 38.49 ± 1.22 ms in the superior region compared with 31.91 ± 1.58 ms in the inferior region (Fig. S2, F–J). The full duration of Mode 1 mito-iATP transients in the superior region was 157.5 ± 2.7 ms, similar to that in the inferior region (155.40 ± 4.13 ms). Mode 2 mito-iATP durations were longer in the superior region (282.20 ± 16.74 ms) than in the inferior region (240.0 ± 8.05 ms; P = 0.046). Given that the recovery phase of Mode 2 events reflects oxidative phosphorylation capacity, the observed ∼240- to 282-ms recovery window suggests that dip-dominant cells can sustain firing only up to ∼3.5–4.2 Hz, as faster pacing would limit ATP replenishment before the next beat.

The mito-iATP signal mass rate (i.e., total signal mass per trace ÷ recording time) of Mode 1 sites was significantly larger in superior myocytes (704.0 ± 76.92 AU) than inferior myocytes (292.80 ± 28.17 AU), indicating greater mitochondrial ATP production in the leading pacemaker zone (Fig. 3 D; P = 0.0010). Mode 1 mito-iATP amplitudes in the superior region (1.28 ± 0.02 F/F_0_) were similar to those in the inferior region (1.25 ± 0.02 F/F_0_) (Fig. 3 E; P = 0.98). The frequency of Mode 1 mito-iATP transients in the superior region ranged from 1.2 to 4.5 Hz, with a mean frequency of 3.51 ± 0.28 Hz. In the inferior node, mito-iATP transient frequency never exceeded 2.60 Hz, and exhibited a mean frequency of 2.05 ± 0.22 Hz (Fig. 3 F; P = 0.0173).

The integrated ATP deficit rate (i.e., negative signal mass) during Mode 2 events was significantly larger in superior myocytes (−775.90 ± 86.70 AU) than inferior myocytes (−383.10 ± 37.46 AU) (Fig. 3 G; P = 0.0023), indicating greater mitochondrial ATP consumption in the leading pacemaker zone. Dip amplitudes were virtually identical between regions (0.77 ± 0.01 vs. 0.79 ± 0.02 F/F_0_; Fig. 3 H; P = 0.99). Mean Mode 2 event frequencies in superior and inferior nodes were 3.27 ± 0.17 Hz and 1.91 ± 0.23 Hz, respectively (Fig. 3 I; P = 0.0056), the latter of which is well below the ∼3.5- to 4.2-Hz ceiling predicted from the 240- to 282-ms recovery window.

Thus, in Mode 2 microdomains, ATP is drawn out of the matrix faster than it is produced during the action potential, generating a brief deficit that is repaid more slowly during diastole. Because Mode 1 (gain) and Mode 2 (dip) regions are spatially distinct, this imbalance implies that high-demand microdomains (Mode 2) temporarily outstrip the surplus generated in high-production zones (Mode 1). Matrix diffusion and a diastolic repayment window, therefore, appear essential for beat-to-beat energetic homeostasis in pacemaker cells.

Brief exposure to FCCP (1 µM), an uncoupler of oxidative phosphorylation, abolished both waveform types in every cell examined (Fig. 3, B–I). Thus, beat-locked mitochondrial ATP transients depend on an intact proton-motive force and, therefore, on ongoing oxidative phosphorylation. The complete suppression of Mode 1 signals—and the greater reduction in Mode 2 amplitude—in superior myocytes underscores their heavier reliance on oxidative phosphorylation and highlights the tight electrometabolic coupling that supports the dominant pacemaker site. In contrast, the data suggest that inferior mitochondria make only a modest contribution to the nodal cytosolic ATP pool under basal conditions.

Intact-node multiphoton imaging can be confounded by residual micromotion, focus drift, and scan registration error, even in the presence of blebbistatin. Accordingly, to verify that beat-locked mito-iATP fluorescence changes reflect genuine ATP dynamics rather than optical artifacts, we implemented an ATP-insensitive reference reporter acquired simultaneously through the same optical path (Fig. S3). We expressed mito-iATP fused to a HaloTag protein and labeled with the red-shifted ligand Janelia Fluor JFX554, recording both channels concurrently. Beat-locked oscillations were exclusively present in the mito-iATP channel, whereas the HaloTag-JFX554 reference remained stable (Fig. S3, A and C).

To quantitatively assess the contribution of potential motion artifacts, we analyzed the relationship between the raw baseline-normalized fluorescence (ΔF/F_0_) and the ratiometric signal (ΔR/R_0_). The rationale for this analysis is that if out-of-focus motion or tissue drift were driving the observed transients, both the sensor and reference channels would fluctuate proportionately. Ratiometric division (R/R_0_ = (F_mito-iATP_/F_mito-iATP,0_)/(F_HaloTag_/F_HaloTag,0_)) would therefore cancel out these shared optical artifacts, resulting in a ΔR/R_0_ amplitude significantly smaller than the raw ΔF/F_0_. Conversely, if the signal is driven purely by ATP binding, dividing by the stable reference channel will yield a ratiometric amplitude identical to the raw signal. By plotting the peak amplitudes of ΔF/F_0_ against ΔR/R_0_ for mito-iATP events, we observed a robust, near 1:1 linear relationship for Mode 1 (gain; slope = 1.07, R^2^ = 0.92) and Mode 2 (dip; slope = 1.09, R^2^ = 0.97) transients (Fig. S3, B and D).

While this 1:1 correlation suggests that motion artifacts are negligible under standard conditions, we sought to explicitly validate that our HaloTag-based approach is sensitive to optical perturbations when they do occur. As a positive control, we imposed a controlled axial defocus (z-step) in intact SA node tissue during multiphoton acquisition (Fig. S3 E). This z-step produced a robust excursion in the HaloTag reference channel, demonstrating that the reference readout reliably reports optical-path perturbations. Importantly, the defocus-induced distortion in the mito-iATP channel was strongly suppressed by baseline-normalized ratiometry (R/R_0_) (Fig. S3 F), confirming that this strategy successfully detects and mitigates motion and focus artifacts *in situ*. Together, the demonstrated sensitivity of the reference channel to imposed defocus, combined with the tight 1:1 raw-to-ratiometric correlation observed during normal acquisitions, strongly suggests that motion and focus artifacts contribute negligibly to our recordings, and that the observed beat-to-beat signals represent bona fide ATP dynamics.

### Uncoupling oxidative phosphorylation suppresses electrical excitability and abolishes beat-locked cyto-iATP signals

**Figure 4.**
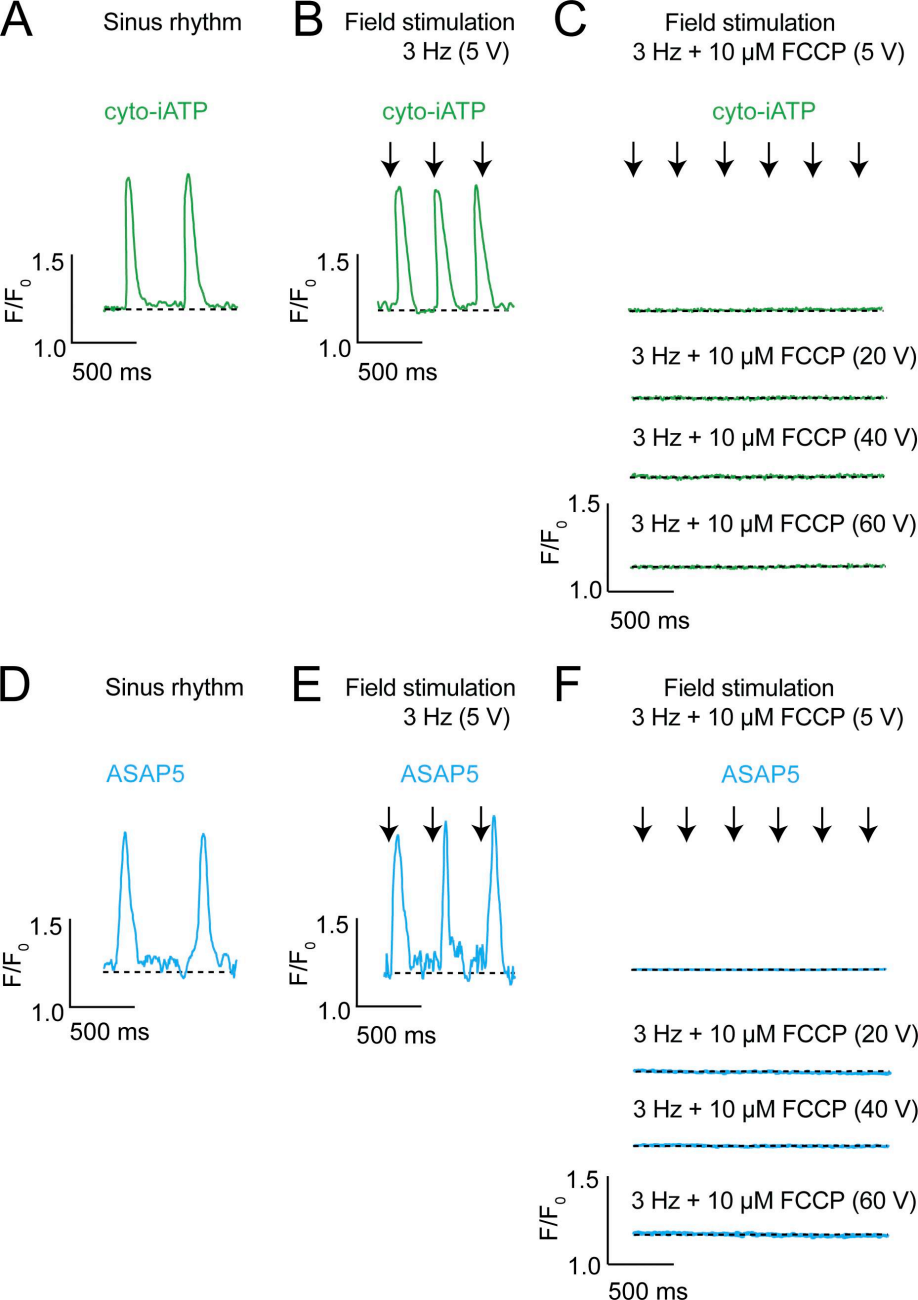
**Mitochondrial ATP synthesis is obligatory for SA node excitability and cannot be bypassed by electrical pacing. (A–F)** Representative recordings of cytosolic ATP dynamics (A–C; cyto-iATP, green) and membrane voltage (D–F; ASAP5, blue) obtained from intact SA node preparations. **(A and D)** Beat-locked cyto-iATP transients with positive deflections and corresponding voltage signals recorded during spontaneous sinus rhythm. **(B and E)** Entrainment of cyto-iATP and voltage signals during field stimulation at 3 Hz (5 V). **(C and F)** Loss of excitation–metabolism coupling during mitochondrial uncoupling with FCCP (10 µM). Traces were acquired during continued pacing at 3 Hz with stepwise increases in stimulation amplitude (5–60 V). For ASAP5 recordings, upward deflections correspond to membrane depolarization. Traces are shown as normalized fluorescence (F/F_0_). The dashed line indicates baseline fluorescence (F/F_0_ = 1). Scale bars: 0.5 F/F_0_, 500 ms.

To determine whether the loss of beat-locked ATP signals during mitochondrial uncoupling reflects direct disruption of ATP production (rather than an optical artifact) or instead represents a failure of electrical activation, we compared cyto-iATP dynamics with a parallel optical readout of membrane voltage (Fig. 4). In spontaneously active SA node myocytes, cyto-iATP exhibited rhythmic transients during sinus rhythm (Fig. 4 A). Imposing field stimulation (3 Hz, 5 V) entrained cyto-iATP transients, indicating that cytosolic ATP signals can be driven by externally paced excitation (Fig. 4 B). In the presence of the mitochondrial protonophore FCCP (10 µM), cyto-iATP transients were abolished during 3-Hz stimulation at 5 V and remained absent even when stimulus intensity was increased stepwise (20–60 V) (Fig. 4 C), indicating that mitochondrial uncoupling prevents electrically evoked ATP responses.

Next, using the novel genetically encoded voltage indicator, ASAP5 (Hao et al., 2024), we tested whether FCCP also disrupts the underlying electrical response to field stimulation (Fig. 4, D–F). ASAP5 signals were rhythmic during sinus rhythm and were robustly entrained by field stimulation at 5 V (Fig. 4, D and E). Strikingly, stimulus-locked ASAP5 voltage signals were abolished by FCCP and were not restored by increasing stimulus intensity up to 60 V (Fig. 4 F). Thus, under our conditions, FCCP renders SA node myocytes electrically unresponsive to field stimulation, providing a parsimonious explanation for the simultaneous loss of beat-locked cyto-iATP signals. Together, these data support the conclusion that intact mitochondrial proton-motive force is required to sustain the excitation–metabolism coupling necessary for beat-locked cytosolic ATP dynamics in pacemaker myocytes.

### Mitochondrial Ca^2+^ uptake and ANT-dependent exchange support beat-locked ATP transients

**Figure 5.**
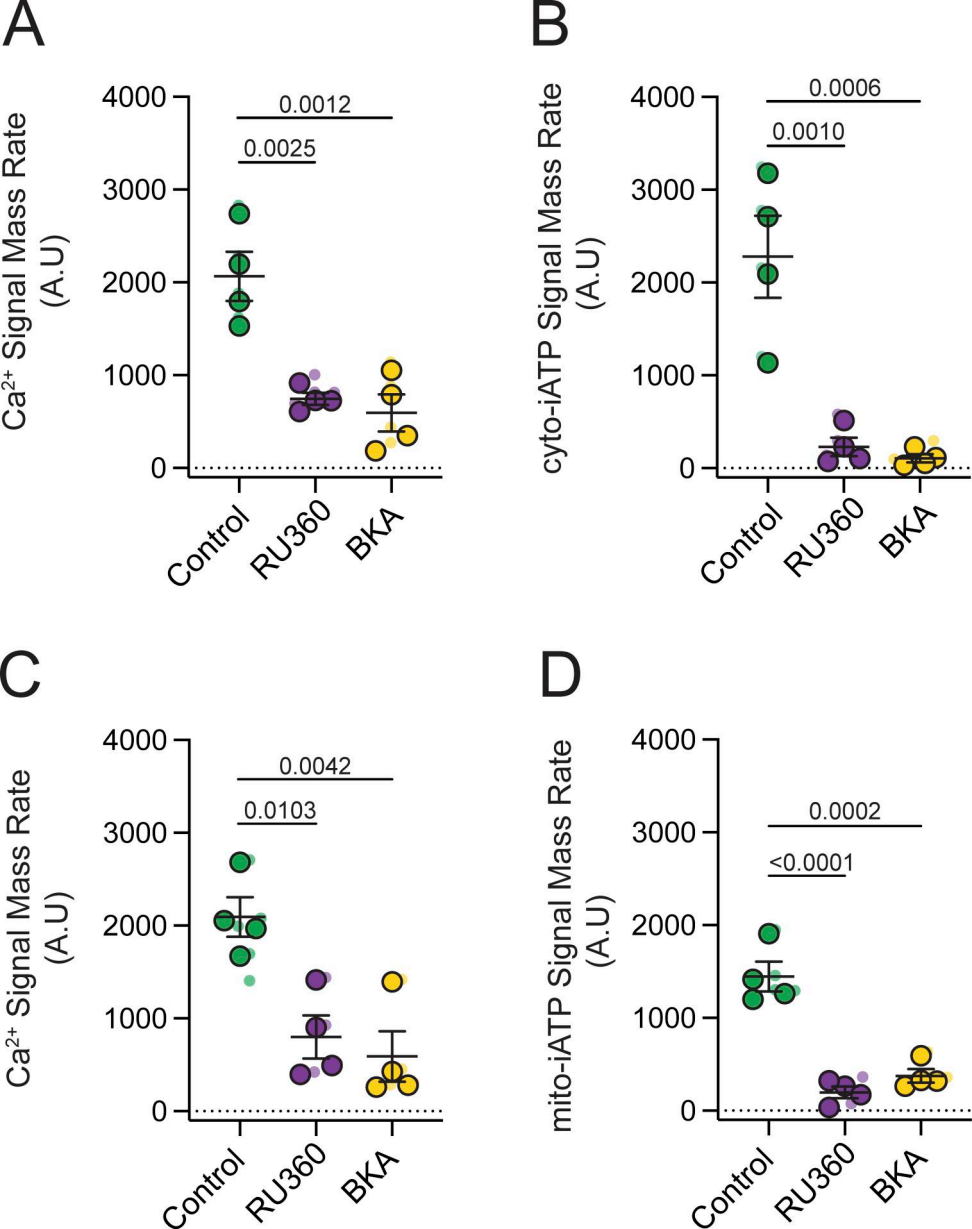
**Mitochondrial Ca** ^**2+**^ **uptake and ANT-mediated ATP export are required for beat-to-beat metabolic signaling. (A and B)** Summary data from SA node myocytes expressing the cytosolic ATP sensor (cyto-iATP). **(A)** Intracellular Ca^2+^ signal mass rates measured under control conditions, after inhibition of the MCU with RU360 (5 µM) and following blockade of the ANT with BKA (10 µM). **(B)** Corresponding cytosolic ATP signal mass rates obtained under the same conditions. **(C and D)** Summary data from parallel experiments in SA node myocytes expressing the mitochondrial ATP sensor (mito-iATP). **(C)** Intracellular Ca^2+^ signal mass rates. **(D)** Mitochondrial ATP signal mass rates. Data are presented as means ± SEM (*N* = 4 mice per group). P values are shown above comparisons. Large circles denote per-animal means; small circles indicate individual biological replicates. *N* represents the number of independent mice.

To probe the sequence linking Ca^2+^ release to ATP production and delivery, we perturbed mitochondrial Ca^2+^ entry and ATP/ADP exchange across the inner mitochondrial membrane (Fig. 5). To inhibit the MCU, we used RU360 (5 µM), as prior work demonstrates that extracellular RU360 effectively permeates the sarcolemma of intact cardiomyocytes to specifically block mitochondrial Ca^2+^ uptake (Matlib et al., 1998; Kim et al., 2011). We found that a 30-min incubation with RU360 modestly reduced cytosolic Ca^2+^ transients (Fig. 5 A), nearly eliminated cyto-iATP transients (Fig. 5 B), attenuated mitochondrial Ca^2+^ transients (Fig. 5 C), and abolished Mode 1 mito-iATP gains (Fig. 5 D).

We next inhibited adenine nucleotide exchange using bongkrekic acid (BKA), a canonical inhibitor of the ANT (Henderson and Lardy, 1970; Ruprecht et al., 2019). BKA (10 µM) modestly reduced cytosolic Ca^2+^ transients (Fig. 5 A), abolished cyto-iATP transients (Fig. 5 B), disrupted mitochondrial Ca^2+^ signals (Fig. 5 C), and abolished mito-iATP transients (Fig. 5 D). These results provide an independent perturbation consistent with the conclusion that beat-locked ATP oscillations in the cytosol require intact mitochondrial ATP production and export; when ANT-mediated exchange is inhibited, neither glycolysis nor phosphocreatine buffering is sufficient to preserve beat-locked ATP pulses. Together, these pharmacological perturbations are consistent with a model in which mitochondrial Ca^2+^ entry and ANT-dependent nucleotide exchange act in series to enable the generation and cytosolic delivery of beat-locked ATP transients in SA node myocytes.

### Regional coupling of beat-locked ATP dynamics to mitochondrial redox state in the intact SA node

**Figure 6.**
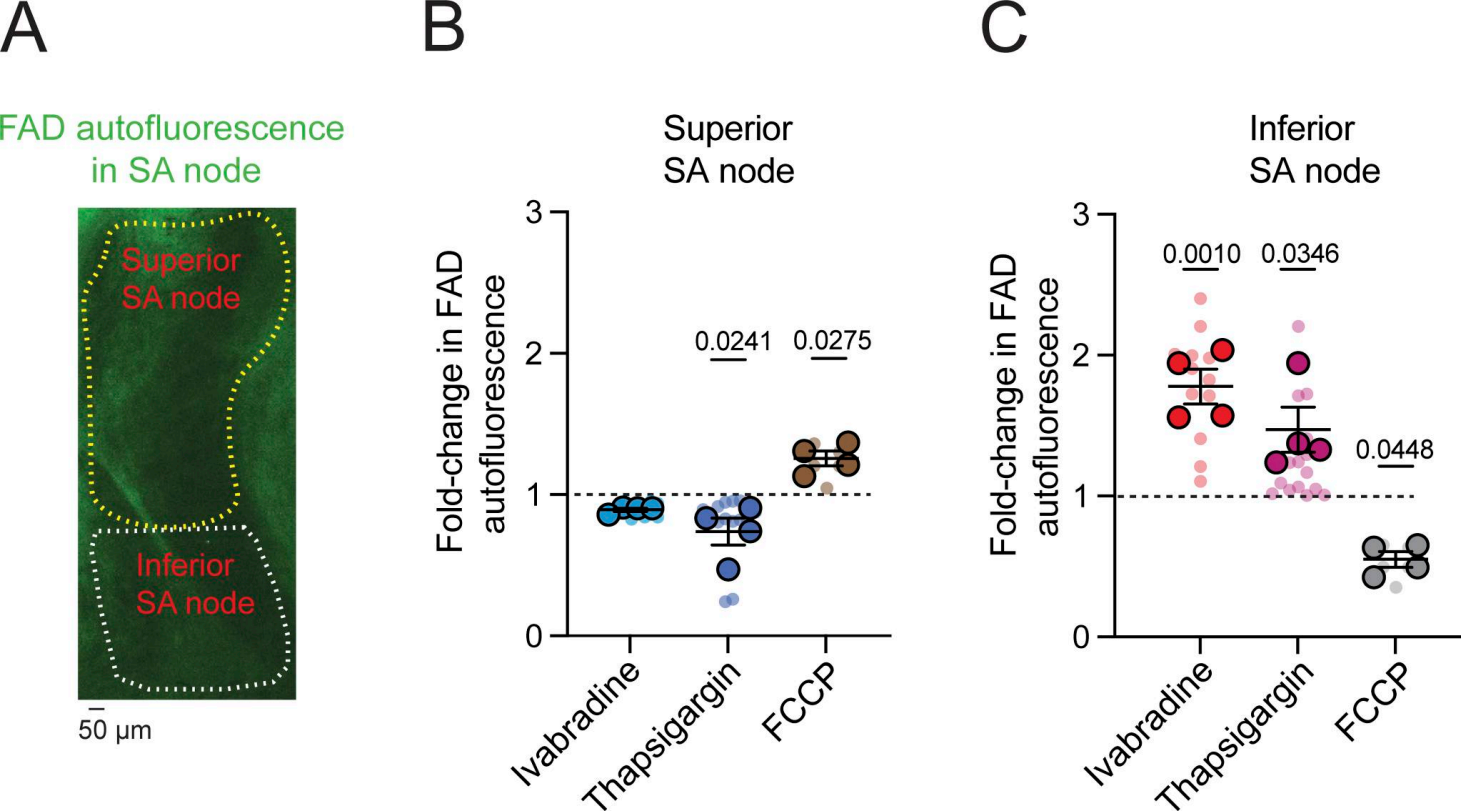
**Distinct mitochondrial redox regulation in the superior versus inferior SA node. (A)** Representative FAD autofluorescence image of a mouse SA node illustrating spatial segregation of the superior (yellow outline) and inferior (white outline) regions. Scale bar, 50 µm. **(B and C)** Quantification of normalized FAD autofluorescence (F/F_0_) in the superior (B) and inferior (C) SA node under control conditions and following I_f_ inhibition with ivabradine (30 µM), SERCA inhibition with thapsigargin (1 µM), or mitochondrial uncoupling with FCCP (1 µM). The dashed line indicates baseline fluorescence (F/F_0_ = 1). P values are shown above comparisons. P values indicate comparisons relative to baseline (F/F_0_ = 1). Data are presented as means ± SEM (*N* = 4 mice). Large circles denote per-animal means; small circles indicate individual biological replicates. *N* represents the number of independent mice.

To integrate the superior–inferior gradients in cyto- and mito-iATP with a calibration-independent readout of mitochondrial redox control, we measured endogenous FAD autofluorescence by two-photon microscopy in intact SA node preparations while applying the same perturbations used for ATP imaging (ivabradine, thapsigargin, FCCP) (Fig. 6). ROIs were defined anatomically as superior and inferior SA node domains (Fig. 6 A). Because oxidized flavoproteins are fluorescent and these signals are quenched by reduction, increases in FAD autofluorescence report net oxidation of the mitochondrial flavin pool and decreases report net reduction (Huang et al., 2002; Berthiaume et al., 2019).

In the superior SA node, where the cyto-iATP signal mass rate and calibrated diastolic [ATP]_i_ were highest, ivabradine (30 µM) did not significantly alter FAD autofluorescence relative to baseline, whereas thapsigargin (1 µM) produced a significant downward shift, consistent with a net reduction, and FCCP (1 µM) induced the expected oxidizing shift (Fig. 6 B). Thus, relative to baseline, thapsigargin decreased FAD autofluorescence (P = 0.0241), while FCCP significantly increased FAD (P = 0.0275).

In contrast, in the inferior SA node, all three interventions significantly altered FAD autofluorescence relative to baseline, both in opposing directions (net oxidation) (Fig. 6 C). Ivabradine increased FAD autofluorescence (P = 0.0010), thapsigargin also increased FAD (P = 0.0346), whereas FCCP decreased FAD autofluorescence relative to baseline (P = 0.0448).

Together, the region-resolved FAD responses (Fig. 6, B and C) align with the ATP data by indicating that superior and inferior pacemaker zones occupy distinct energetic control regimes: the superior region sustains higher beat-locked ATP throughput and responds to ivabradine or thapsigargin with a net reduction of the flavin pool, consistent with greater energetic reserve, whereas the inferior region exhibits lower ATP availability and shows net flavin oxidation under the same perturbations, consistent with tighter supply–demand constraint. Thus, the opposing FAD shifts in superior versus inferior regions provide a calibration-independent redox signature that reinforces the superior-to-inferior energetic gradient revealed by both cyto-iATP and mito-iATP measurements. We next asked whether this functional and redox hierarchy is underpinned by a corresponding gradient in mitochondrial capacity.

### Regional differences in mitochondrial abundance define distinct SA node myocyte phenotypes

**Figure 7.**
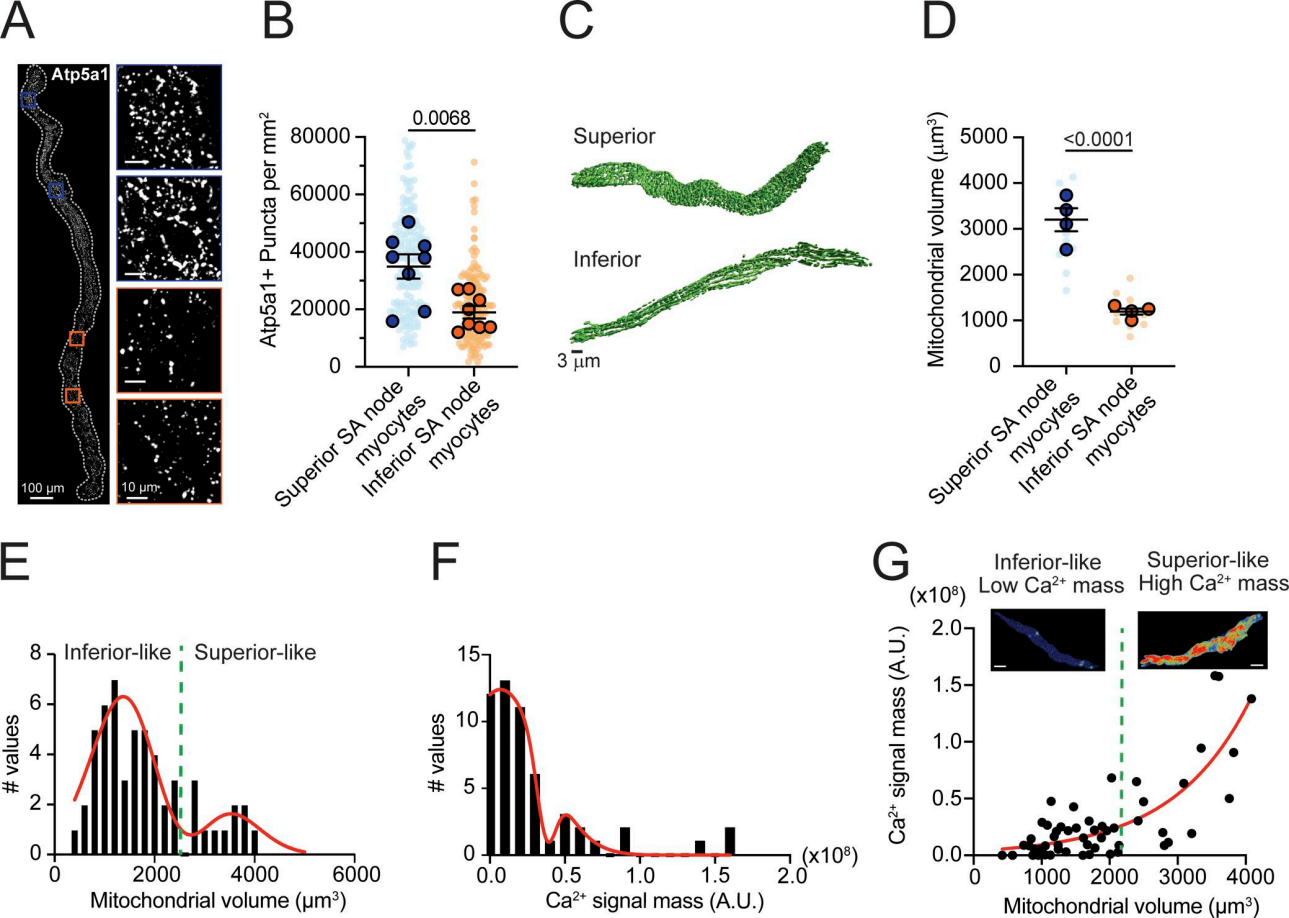
**Regional heterogeneity in mitochondrial volume and ATP5A1 expression in SA node myocytes. (A)** Representative RNAscope *in situ* hybridization image of a whole-mount SA node labeled for ATP5A1 mRNA (white). Insets show higher magnification views of ATP5A1 puncta in superior (blue outlines) and inferior (orange outlines) regions. Scale bars: 100 µm (whole mount), 10 µm (insets). **(B)** Quantification of ATP5A1 punctate density in superior and inferior SA node myocytes. **(C)** Representative three-dimensional surface renderings of the mitochondrial reticulum (MitoTracker Green) in myocytes isolated from superior (top) and inferior (bottom) regions. **(D)** Quantification of total mitochondrial volume per myocyte (*N* = 4 mice per group). **(E and F)** Frequency distributions of mitochondrial volume (E) and integrated Ca^2+^ signal mass (F) pooled across all analyzed myocytes. Red curves indicate Gaussian fits, resolving two populations. The green dashed line (≈2.2 × 10^3^ µm^3^) denotes the intersection used to classify low- and high-volume/mass phenotypes. **(G)** Relationship between mitochondrial volume and Ca^2+^ signal mass for individual myocytes. The red curve represents an exponential fit (R^2^ = 0.73). Insets show maximum-intensity projections of representative Ca^2+^ transients from low- and high-volume groups. Scale bars, 5 µm. P values are shown above comparisons. Large circles denote per-animal means; small circles indicate individual biological replicates. *N* represents the number of independent mice.

The opposing FAD redox shifts in superior versus inferior regions provide a calibration-independent signature that reinforces the energetic gradient revealed by cyto-iATP and mito-iATP. A potential explanation for these coupled functional differences is a structural one: the superior SA node may be endowed with greater mitochondrial capacity, enabling higher beat-locked ATP throughput and a distinct redox operating point. Thus, we next asked whether this functional and redox hierarchy is underpinned by a corresponding gradient in mitochondrial capacity, testing the hypothesis that mitochondrial abundance scales with the superior–inferior energetic hierarchy. To this end, we quantified mitochondrial content, first in intact-node whole mounts and then in anatomically identified single myocytes (Fig. 7).

To assess whether regional energetic differences have a structural basis, we used *in situ* hybridization (RNAscope) to measure ATP5A1 mRNA expression in whole-mount SA node preparations. ATP5A1 encodes the catalytic α-subunit of F_1_F_o_-ATP synthase (Walker, 2013; Kühlbrandt, 2019), the enzyme responsible for mitochondrial ATP production. The superior region exhibited visibly denser ATP5A1 labeling than the inferior region (Fig. 7 A). Quantification confirmed a significantly higher density of ATP5A1-positive puncta in the superior domain (P = 0.0068; Fig. 7 B). This mRNA gradient suggests that superior SA node cells are transcriptionally programmed to produce more ATP synthase, potentially supporting greater oxidative phosphorylation capacity. To complement this molecular analysis, we performed volumetric 3D reconstructions of region-identified, isolated SA node myocytes labeled with MitoTracker Green to assess total mitochondrial mass. Superior cells possessed substantially larger mitochondrial networks than inferior cells (Fig. 7 C), with a greater than twofold increase in total mitochondrial volume (3,014 ± 245 µm^3^ vs. 1,161 ± 121 µm^3^; P <0.0001; Fig. 7, C and D). Thus, superior SA node myocytes benefit from both increased mitochondrial mass and elevated ATP5A1 expression, suggesting convergent structural and biochemical mechanisms underlying their high-gain ATP phenotype.

To determine whether mitochondrial size constrains the Ca^2+^ burden of individual pacemaker cells, we analyzed the full pool of SA node myocytes instead of presorting them by anatomical origin. Pooling was justified for three reasons: (1) 3D reconstructions revealed a clearly bimodal distribution of mitochondrial volumes—small-volume cells came almost exclusively from the inferior node and large-volume cells from the superior node—so treating the tissue as two rigid classes would have narrowed the quantitative range available for correlation analysis and reduced power. (2) Ca^2+^ transients fire at ∼4 Hz in superior myocytes versus ∼1 Hz in inferior cells (Grainger et al., 2021); because transient amplitudes are comparable, frequency alone accounts for their greater total Ca^2+^ flux. In our dataset, every Ca^2+^ record was normalized to its own firing rate, removing this regional bias and allowing us to test whether bigger mitochondrial networks support larger beat-normalized Ca^2+^ loads across the entire spectrum of phenotypes. (3) The limited yield of viable, dye-loaded cells obtained from finely dissected subregions—and the need to collect both 3D mitochondrial stacks and high-speed Ca^2+^ movies—would have left separate regional datasets underpowered. Pooling therefore maximizes sample size and provides an unbiased, statistically robust test of structure–function coupling.

3D mitochondrial stacks, obtained by SRRF imaging of the mitochondrial reticulum, and movies of cytosolic Ca^2+^ signals were generated from acutely dissociated SA node myocytes loaded with MitoTracker and Fluo-4 AM. Mitochondrial volume histograms displayed two well-separated peaks; a double-Gaussian fit yielded a threshold of ∼2,200 µm^3^ that segregated “inferior-like” (low-volume) from “superior-like” (high-volume) cells (Fig. 7 E). An analogous bimodal pattern was observed for Ca^2+^ signal mass (Fig. 7 F). Across the pooled population, mitochondrial volume was a strong predictor of Ca^2+^-handling load: larger mitochondria supported proportionally greater Ca^2+^ signal mass, with an exponential fit explaining 73% of the variance (Fig. 7 G). Ca^2+^ signal mass scaled with mitochondrial volume, such that cells with lower mitochondrial content failed to reach the Ca^2+^ output observed in high-volume cells, suggesting that organelle size constrains the upper limit of signaling capacity.

Together with the higher beat-locked cyto- and mito-iATP throughput in the superior node (see Figs. 1, 2, and 3, above) and the faster intrinsic firing previously reported in superior versus inferior SA node myocytes (Grainger et al., 2021), these data indicate that superior pacemaker cells combine an expanded mitochondrial network with stronger Ca^2+^ signaling and higher energetic throughput, whereas inferior cells couple reduced mitochondrial content to lower Ca^2+^-linked demand and lower ATP turnover. Thus, regional differences in mitochondrial volume represent a structural determinant that helps define distinct metabolic and electrophysiological phenotypes within the SA node (Santana and Earley, 2026).

### Beat-locked Ca^2+^ transients trigger cytosolic ATP increases in SA node myocytes

**Figure 8.**
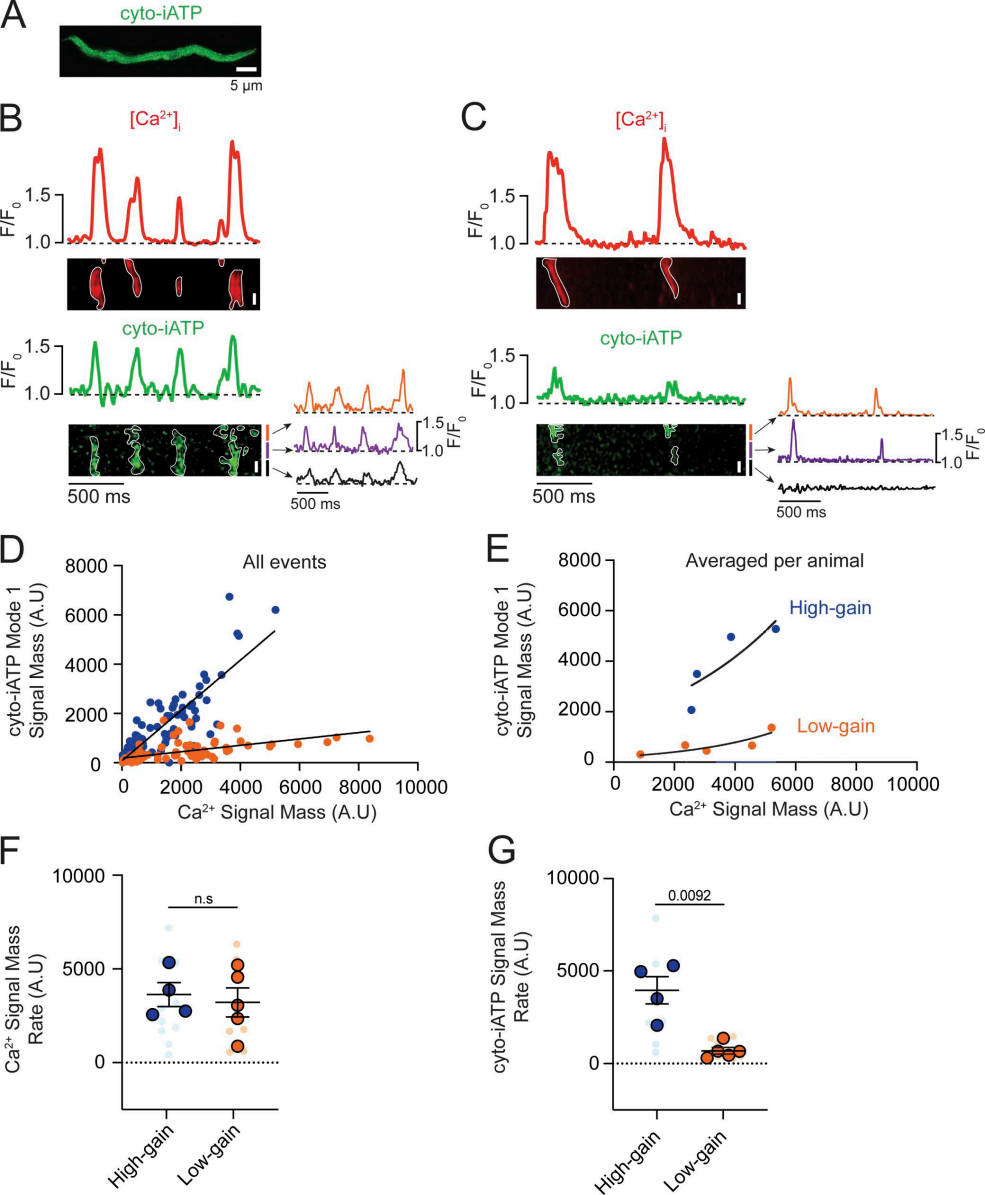
**Beat-to-beat ATP synthesis is coupled to intracellular Ca** ^**2+**^ **release through distinct high- and low-gain metabolic transfer functions in SA node myocytes. (A)** Representative confocal image of an isolated SA node myocyte expressing the cytosolic ATP sensor (cyto-iATP). Scale bar, 5 µm. **(B and C)** Simultaneous confocal line-scan recordings of intracellular Ca^2+^ (red, top) and cyto-iATP (green, bottom). The dashed line indicates baseline fluorescence (F/F_0_ = 1). **(B)** Example of a myocyte classified as high-gain, showing temporally aligned Ca^2+^ transients and cyto-iATP signals. Traces extracted from local ROIs (colored traces) illustrate spatially resolved Ca^2+^ and ATP dynamics. **(C)** Example of a myocyte classified as low-gain, showing Ca^2+^ transients with minimal corresponding cyto-iATP signals. **(D)** Event-level relationship between Ca^2+^ signal mass (input) and cyto-iATP signal mass (output). Data are separated into two populations: high-gain events (blue circles) and low-gain events (orange circles). Solid lines indicate linear fits for each population. **(E)** Cell-averaged relationship between Ca^2+^ and cyto-iATP signal mass, preserving bimodal separation between high-gain (blue) and low-gain (orange) groups. **(F and G)** Summary quantification of Ca^2+^ signal mass rate (F) and cyto-iATP signal mass rate (G) for high- and low-gain populations. P values are shown above comparisons. Large circles denote per-animal means; small circles indicate individual biological replicates.

Having shown that mitochondrial volume scales with the Ca^2+^ burden that a cell must service, we next asked whether the timing of Ca^2+^ release likewise governs the beat-to-beat rise of ATP in the cytosol, turning structural capacity into real-time metabolic output. To do this, we loaded dissociated SA node myocytes expressing cyto-iATP with the red-shifted Ca^2+^ indicator, Rhod-3, and simultaneously tracked cytosolic ATP and Ca^2+^ using a confocal microscope in line-scan mode (Fig. 8 A).

Simultaneous line-scan imaging of Ca^2+^ release and cyto-iATP fluorescence in two representative pacemaker cells with high (Fig. 8 B) and low (Fig. 8 C) frequency of Ca^2+^-release events showed that in both cases, every Ca^2+^-release event is paired with a rise in cytosolic ATP, confirming beat-locked metabolic coupling. The spatial extent of the ATP rise was found to differ markedly: in the high-frequency cell (Fig. 8 B), the increase was spread across a much broader swath of the cytoplasm, whereas in the low-frequency cell (Fig. 8 C), the increase was restricted to a narrow segment. Quantitative differences in these patterns, exemplified by traces from three ROIs shown beneath each cyto-iATP image, highlight localized versus widespread ATP transients.

Given the variations in spatial distributions, we quantified the signal mass of Ca^2+^ signals and their associated cyto-iATP events. As shown in Fig. 8, D and E, pooled events from all cells imaged segregated into two groups, both of which could be fit to linear functions distinguished by the different slopes of their Ca^2+^-ATP relationships: 1.02 Δcyto-iATP/ΔCa^2+^ for high-gain events and 0.12 Δcyto-iATP/ΔCa^2+^ for low-gain events (P <0.0001). Notably, plotting averaged cyto-iATP and Ca^2+^ signal mass per cell segregated the data into two clear gain regimes: a low-gain group with minimal ATP amplification and a high-gain group with steep, exponential Ca^2+^-to-ATP coupling (Fig. 8 E).

Despite the similarity of Ca^2+^ signal mass rate (Fig. 8 F) across phenotypes, cyto-iATP signal mass rate was significantly larger in high-gain cells (P = 0.0092), underscoring the higher energetic output in these cells over time (Fig. 8 G). This dichotomy mirrors the intact node, where superior myocytes exhibit greater signal mass rates than inferior myocytes; it is further reinforced by higher event frequencies in high-gain cells (2.56 ± 0.16 Hz) versus low-gain cells (1.50 ± 0.18 Hz; P = 0.003) that closely match regional values in tissue (Figs. 1 and 2).

A kinetic analysis of Ca^2+^ transients showed slower dynamics in high-gain cells, with a longer decay time (t_1/2_ = 92.48 ± 20.77 ms; P = 0.0419) and prolonged duration (434.70 ± 50.83 ms; P = 0.0125) compared with low-gain cells (t_1/2_ = 41.11 ± 8.70 ms; 247.40 ± 33.71 ms) (Fig. S4, A–E). In contrast, cyto-iATP transients exhibited comparable rise times in high-gain (99.81 ± 10.56 ms) and low-gain (90.88 ± 11.30 ms) cells, as well as similar decay times (t_1/2_ ∼47–66 ms) and durations (∼247.4–286.1 ms) (Fig. S4, F–H). Thus, cytosolic ATP output in pacemaker myocytes is not simply proportional to Ca^2+^ load but instead falls into discrete high- and low-gain modes, echoing the superior–inferior metabolic hierarchy of the intact SA node.

### High- and low-gain mitochondrial ATP production in SA node myocytes

**Figure 9.**
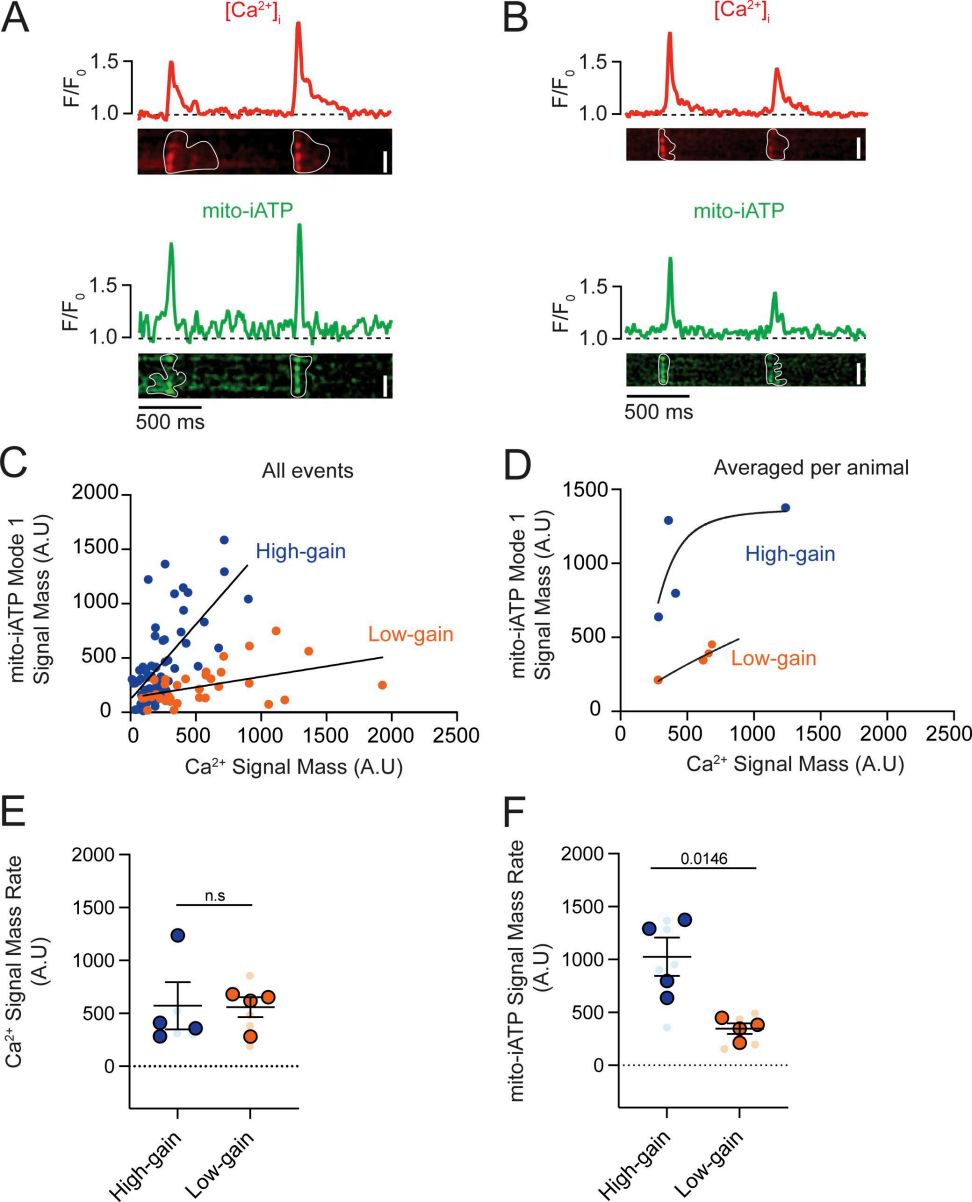
**Beat-to-beat mitochondrial ATP synthesis exhibits bimodal coupling to intracellular Ca** ^**2+**^ **release in SA node myocytes. (A and B)** Representative confocal line-scan recordings showing simultaneous intracellular Ca^2+^ transients (red, top) and mitochondrial ATP signals (mito-iATP; green, bottom). The dashed line indicates baseline fluorescence (F/F_0_ = 1). **(A)** Example of a myocyte classified as high-gain, displaying temporally aligned Ca^2+^ transients and mito-iATP signals. **(B)** Example of a myocyte classified as low-gain, in which Ca^2+^ transients are accompanied by minimal mito-iATP responses. **(C)** Event-level relationship between Ca^2+^ signal mass (input) and mito-iATP signal mass (output). Data are segregated into two populations: high-gain events (blue circles) and low-gain events (orange circles), with solid lines indicating linear fits. **(D)** Cell-averaged relationship between Ca^2+^ and mito-iATP signal mass, fitted with a Hill function for each group (high-gain, blue; low-gain, orange). **(E and F)** Summary quantification of Ca^2+^ signal mass rate (E) and mito-iATP signal mass rate (F) for high- and low-gain populations. P values are shown above comparisons. Data are presented as means ± SEM (*N* = 4 mice per group). Large circles denote per-animal means; small circles indicate individual biological replicates. *N* represents the number of independent mice.

We found that Mode 1 events—rapid postbeat rises in mito-iATP—are tightly beat-locked, with Ca^2+^ transients preceding ATP onset by <50 ms, consistent with rapid SR-to-mitochondrial Ca^2+^ transfer gating oxidative phosphorylation. The magnitude of ATP elevation per unit Ca^2+^ load varied sharply between cells. Representative high-gain cells (Fig. 9 A) produced broad, high-amplitude ATP increases spanning most of the scan region, while low-gain cells (Fig. 9 B) generated smaller, spatially restricted rises, despite comparable Ca^2+^ signals.

Pooling the signal mass of all associated Ca^2+^ and mito-iATP events across cells yielded an apparently piecewise-linear Ca^2+^-ATP relation with two regimes: low slope (0.19 Δmito-iATP/ΔCa^2+^) and high slope (1.38 Δmito-iATP/ΔCa^2+^) (Fig. 9 C); but because events are nested within cells, this marginal pattern reflects aggregation. Accordingly, per-cell fits showed Hill models with greater apparent cooperativity in high-gain cells (Hill coefficient, 2.88) than low-gain cells (Hill coefficient, 0.78) and distinct plateaus (Fig. 9 D). The sigmoidal behavior is expected from thresholded, cooperative mitochondrial Ca^2+^ control, where MICU1/2 gatekeeping of the MCU sets the Ca^2+^ threshold and gain (Mallilankaraman et al., 2012; Csordas et al., 2013; Kamer et al., 2017). Plateau differences imply capacity differences: the lower maximum output (ceiling) in low-gain cells is consistent with reduced mitochondrial mass and/or diminished per-mitochondrion respiratory capacity. It is important to note, however, that because iATPSnFR family sensors have finite dynamic ranges and can saturate at high ATP (Lobas et al., 2019; Marvin et al., 2024; Rhana et al., 2024), the upper plateau in high-gain cells could partly reflect sensor saturation rather than biology, so ceiling differences are interpreted cautiously.

We extended the analysis by calculating Ca^2+^ and mito-iATP signal mass rates (total signal mass per trace divided by recording time), which index Ca^2+^ load and ATP production over time rather than per-event yield. Ca^2+^ signal mass rate did not differ between groups (Fig. 9 E), whereas mito-iATP signal mass rate was higher in high-gain cells (P = 0.0146) (Fig. 9 F). Per-cell averages corroborated the two phenotypes, with high-gain cells firing faster (2.49 ± 0.59 Hz vs. 0.92 ± 0.12 Hz; P = 0.0164). For Ca^2+^ transients, rise times did not differ significantly (106.40 ± 17.28 ms vs. 70.39 ± 7.23 ms), but high-gain cells exhibited slower decay (t_1/2_ = 85.31 ± 15.65 ms vs. 39.63 ± 12.17 ms) and longer duration (421.60 ± 65.36 ms vs. 251.90 ± 46.28 ms) (Fig. S5, A and C–E). Mode 1 Mito-iATP event kinetics was similar between groups (rise time, 94.40 ± 17.39 ms vs. 67.11 ± 14.70 ms; decay t_1/2_, ∼59–64 ms; duration, ∼299.80–301.0 ms) (Fig. S5, B and F–H).

Thus, Mode 1 mitochondrial ATP production is stratified into low- and high-gain phenotypes with shallower versus steeper Ca^2+^-ATP gain; high-gain cells rapidly mobilize ATP once a Ca^2+^ gate is crossed and operate nearer a higher ceiling, whereas low-gain cells exhibit a lower ceiling and approach saturation only at higher Ca^2+^ loads. This functional dichotomy echoes the superior–inferior metabolic hierarchy of the intact SA node, revealing that intrinsic cooperativity shapes not only the magnitude but also the dynamic range of pacemaker cell bioenergetics.

To provide a preparation-matched control for optical artifacts in isolated myocytes, we utilized the same mito-iATP-HaloTag fusion protein labeled with JFX554 (Fig. S6). Dual-channel imaging confirmed that mito-iATP fluorescence localized to the mitochondrial network, while the JFX554-HaloTag reference remained spatially and temporally stable (Fig. S6 A). Consistent with our intact-node observations, beat-locked mito-iATP transients, both Mode 1 gains and Mode 2 dips, occurred without corresponding fluctuations in the HaloTag channel. Consequently, baseline-normalized ratiometry (ΔR/R_0_) fully preserved the waveforms of both modes (Fig. S6, B and D). By plotting the raw peak amplitudes (ΔF/F_0_) against the ratiometric amplitudes (ΔR/R_0_), we observed the same robust, near 1:1 linear relationship for both Mode 1 (slope 0.98, R^2^ = 0.95; Fig. S6 C) and Mode 2 (slope = 0.99, R^2^ = 0.98; Fig. S6 E) events. Because isolated myocytes lie flat against the coverslip and lack the complex 3D architecture and out-of-focus scattering present in whole-tissue preparations, the imposed z-step defocus validation performed in the intact node was unnecessary here. Collectively, the convergence of multiple orthogonal lines of evidence strongly suggests that these beat-locked mito-iATP waveforms reflect genuine metabolic dynamics. A generalized mechanical or optical artifact cannot account for signals that are perfectly preserved during ratiometric reference correction, exhibit anatomically distinct bidirectional waveforms (Mode 1 and Mode 2), and manifest in spatially restricted microdomains. Furthermore, such artifacts cannot explain the selective ablation of ATP transients by targeted mitochondrial inhibitors (e.g., RU360, BKA) in blebbistatin-arrested preparations where the primary mechanical trigger persists. Together, these physiological, pharmacological, and optical controls argue against motion, drift, or focus fluctuations as the source of our signals and instead support our model of beat-to-beat mitochondrial ATP synthesis.

### High- and low-load mitochondrial ATP consumption in SA node myocytes

**Figure 10.**
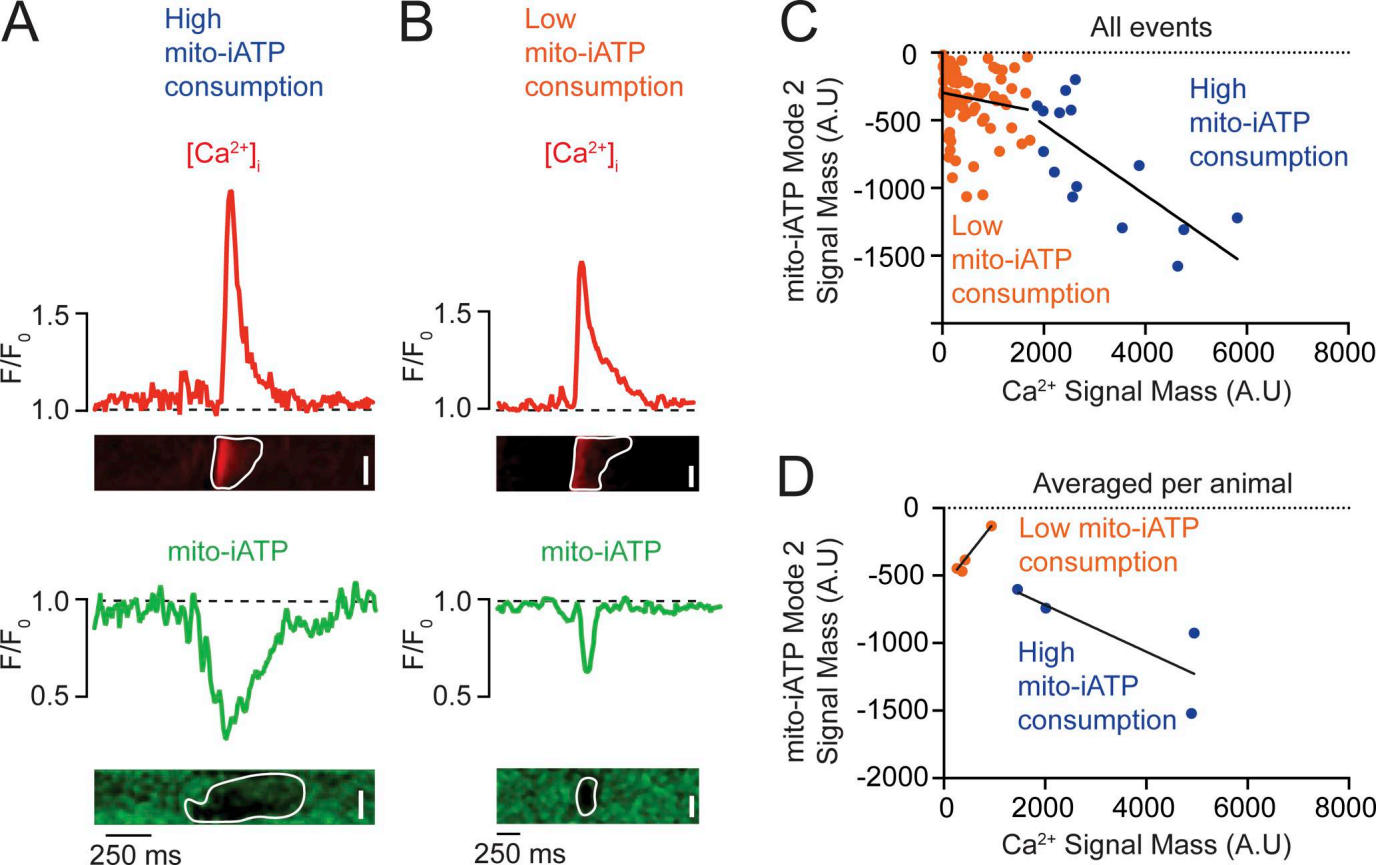
**Beat-to-beat mitochondrial ATP consumption exhibits bimodal coupling to intracellular Ca** ^**2+**^ **release in SA node myocytes. (A and B)** Representative confocal line-scan recordings showing simultaneous intracellular Ca^2+^ transients (red, top) and Mode 2 mitochondrial ATP signals (mito-iATP; green, bottom). The dashed line indicates baseline fluorescence (F/F_0_ = 1). **(A)** Example of a myocyte classified as high-load, in which Ca^2+^ release is associated with large-amplitude negative mito-iATP deflections. **(B)** Example of a myocyte classified as low-load, showing Ca^2+^ transients with smaller accompanying mito-iATP deflections. **(C)** Event-level relationship between Ca^2+^ signal mass (input) and mitochondrial ATP consumption signal mass (output). Data are segregated into two populations: high-load events (blue circles) and low-load events (orange circles). Solid lines indicate linear fits for each population. **(D)** Cell-averaged relationship between Ca^2+^ signal mass and mitochondrial ATP consumption for high-load (blue) and low-load (orange) groups. Scale bars, 5 µm. Data are presented as mean fits (*N* = 4 mice per group). In C, circles represent individual biological events; in D, circles denote per-animal means. *N* represents the number of independent mice.

As was the case in intact SA nodes, we detected Mode 2 events—transient mito-iATP dips following each Ca^2+^ transient, indicating rapid, beat-synchronous ATP depletion in the matrix mitochondria—in isolated myocytes (Fig. 10). Plotting mito-iATP dip signal mass versus Ca^2+^ signal mass (Fig. 10, A and B) revealed two regimes separated by an empirical Ca^2+^ threshold of ∼2,000 AU: below this threshold, events formed a dense, shallow-dip cluster (slope = −0.07 Δmito-iATP/ΔCa^2+^), whereas above it, dips deepened with increasing Ca^2+^ load (slope = −0.26 Δmito-iATP/ΔCa^2+^), as summarized in Fig. 10, C and D. High-load cells drew down ∼twofold more mitochondrial ATP per beat than low-load cells (−905.92 ± 162.30 vs. −407.40 ± 82.41 AU) and fired faster (2.11 ± 0.20 vs. 1.08 ± 0.23 Hz). For Ca^2+^ transients, kinetics did not differ between groups (rise, 80.88–90.01 ms; t_1/2_, 32.88–46.69 ms; duration, 230.90–267.60 ms; Fig. S7, A–E). Similarly, Mode 2 mito-iATP kinetics did not differ between groups (rise, 91.33–107.30 ms; t_1/2_, 39.70–51.21 ms; duration, 232.90–304.60 ms; Fig. S7, F–H).

Thus, mitochondrial ATP consumption is stratified into low- and high-load phenotypes: cells with mito-iATP dips above ∼2,000 AU consume disproportionately more ATP per beat, whereas cells below this threshold consume less despite similar Ca^2+^ inputs, indicating discrete utilization modes that likely contribute to metabolic specialization within the node.

## Discussion

In this study, we demonstrate that ATP production in SA node myocytes is synchronized to each heartbeat rather than being produced continuously and is confined to discrete metabolic microdomains. By imaging mitochondrial ATP alongside Ca^2+^ transients, we observed recurring “ATP gain” peaks and “ATP dip” troughs whose magnitudes and timing matched the electrical cycle, revealing a just-in-time energetic budget tailored to beat-by-beat ionic demand.

### ATP microdomains and regional hierarchy

Regional analyses uncovered pronounced heterogeneity along the node. Superior SA node myocytes, which possess greater mitochondrial volume and reside in regions of higher capillary density, displayed larger ATP gains and higher diastolic [ATP]_i_ than inferior cells, in line with their faster basal firing rates. Pharmacological experiments illuminated SR-to-mitochondrial Ca^2+^ transfer as the proximate trigger for oxidative phosphorylation: thapsigargin abolished ATP oscillations by preventing SR Ca^2+^ refilling, whereas blocking depolarizing HCN channels with ivabradine simply slowed their frequency without affecting their amplitude, demonstrating that the SR Ca^2+^ cycling governs metabolic activation upstream of the I_f_-dependent membrane depolarization.

These structure–function relationships extend earlier anatomical work showing a superior-to-inferior vascular gradient in the mouse node (Grainger et al., 2021). Manning et al. (2024) linked chronic under-perfusion- to hypoxia-inducible factor (HIF)–1α activation, SERCA down-regulation, and reduced PGC-1α–dependent mitochondrial biogenesis. Our results place this hypoxia–HIF cascade within the nodal context, supporting the conclusion that microvascular “wealth” establishes the mitochondrial “capital” available for rapid ATP payouts.

The dominant pacemaking locus within the SA node is also dynamic, migrating along the superior–inferior axis in response to changes in autonomic tone (Yamamoto et al., 1998; Boyett et al., 2000; Brennan et al., 2020). Optical mapping of *ex vivo* rat and human preparations demonstrates the coexistence of two discrete initiation sites—a richly vascularized, mitochondria-dense superior focus and a comparatively hypoperfused inferior focus—that alternate leadership according to sympathetic versus parasympathetic drive (Brennan et al., 2020). Our findings place firm energetic limits on this plasticity: when initiation shifts toward the inferior node, the lower capillary density, reduced mitochondrial reserve, and smaller beat-to-beat ATP gains of this region constrain the maximal rate it can sustain. Thus, although the pacemaker center can relocate, the performance of the newly recruited site remains bounded by the surrounding microvascular and metabolic landscape that we have mapped.

### Metabolic coupling and MCU–ANT axis

Our analysis revealed two coupling modes in pacemaker myocytes. Mode 1 is characterized by beat-locked mitochondrial ATP “gains” and exhibits two phenotypes: a high-gain subtype in which modest Ca^2+^ transients elicit steep ATP increases, and a low-gain subtype with a shallower Ca^2+^-to-ATP coupling slope. Mode 2 shows mitochondrial ATP “dips” that scale linearly and inversely with Ca^2+^ load, consistent with consumption-dominated control. Together, these phenotypes underscore heterogeneity in SR–mitochondrial communication, likely reflecting differences in Ca^2+^-transfer efficacy, mitochondrial reserve, and local substrate availability.

The RU360 and BKA experiments provide direct mechanistic evidence that these ATP modes arise from a bidirectional molecular apparatus rather than passive buffering: both mitochondrial and cytosolic ATP transients are abolished by blocking either MCU or ANT, while Ca^2+^ cycling persists. The elimination of Mode 1 ATP gains by RU360 shows that the gain phase is actively driven by Ca^2+^ entry through the MCU, whereas ANT block suggests that beat-locked ATP pulses vanish if ADP cannot enter—and ATP cannot exit—the matrix. In this framework, the Ca^2+^ clock provides the trigger, while mitochondrial Ca^2+^ entry and nucleotide exchange (via MCU and ANT) support the high-gain metabolic response that sustains beat-locked ATP delivery. When this mitochondrial axis is perturbed, myocytes rely more heavily on basal glycolysis and phosphocreatine buffering, such that Ca^2+^ cycling can persist but becomes energetically weakened. Consistent with this shift, cells transition from a superior-like phenotype that maintains robust oscillatory activity across a broader range of firing frequencies to an inferior-like phenotype in which stable firing is supported over a narrower range.

While our experiments establish the existence of high- and low-gain ATP responses, they do not resolve the underlying molecular determinants. We therefore propose two testable hypotheses for future work: (1) a structure-centric model based on differential SR–mitochondria tethering, and (2) a biochemical framework centered on variable oxidative phosphorylation capacity. In the first case, superior pacemaker cells may express more Mfn2 (or other tethers), narrowing the SR–mitochondrial cleft and amplifying beat-triggered Ca^2+^ transfer. In this scenario, inferior cells, with sparser coupling, would receive smaller mitochondrial Ca^2+^ pulses and thus operate at lower metabolic gain. In the second case, high-gain cells would harbor a denser complex V proteome (e.g., greater ATP5A1 content), allowing faster ATP synthesis per unit Ca^2+^. In this scenario, low-gain cells, with reduced ATP5A1 abundance, would be constrained to shallower slopes. These structural (coupling) and biochemical (flux capacity) gradients together offer a plausible framework for the dual-gain behavior we observe and define a roadmap for future mechanistic dissection.

Our RNAscope analysis provides direct molecular validation of the biochemical hypothesis, revealing significantly higher ATP5A1 mRNA expression in superior versus inferior SA node regions. ATP5A1 encodes the catalytic α-subunit of F_1_F_o_-ATP synthase (Walker, 2013; Kühlbrandt, 2019), and elevated transcript levels likely translate to increased enzyme abundance and enhanced ATP synthetic capacity per mitochondrion. This transcriptional gradient is mechanistically significant for several reasons. First, the ATP5A1 difference may exceed the 2.6-fold mitochondrial volume gradient we observe between superior and inferior cells, indicating convergent structural and biochemical advantages: superior myocytes benefit from both increased mitochondrial mass and higher ATP synthase content per organelle, compounding their energetic capacity. Second, the mRNA gradient reveals transcriptional programming rather than acute metabolic adjustment, implicating upstream regulators such as PGC-1α and estrogen-related receptor α (ERRα)—which are themselves oxygen-sensitive and vascular supply–dependent—as determinants of regional pacemaker identity (Rowe et al., 2010; Scarpulla, 2011). This connects our molecular findings to the vascular heterogeneity reported by Grainger et al. (2021) and suggests that superior cells maintain higher ATP5A1 expression because they receive better oxygen delivery, establishing a feed-forward loop in which vascular supply drives mitochondrial biogenesis and ATP synthase content. Our findings complement recent spatial transcriptomics studies identifying oxidative phosphorylation gene heterogeneity in cardiac conduction system cells (Liang et al., 2021; Kanemaru et al., 2023) by demonstrating that metabolic programming creates functional gradients within SA node regions. Together, these findings establish that the high-gain ATP phenotype in superior cells has a transcriptional basis: enhanced vascular oxygen delivery (Grainger et al., 2021) drives PGC-1α/ERRα-dependent upregulation of ATP5A1 (Rowe et al., 2010; Scarpulla, 2011), amplifying ATP synthetic capacity through both increased mitochondrial mass and elevated ATP synthase content per organelle.

A particularly strong constraint emerged from the FCCP-ASAP5 experiments: when oxidative phosphorylation was uncoupled, stimulus-locked ASAP5 voltage signals were abolished, and stepwise increases in field-stimulation intensity did not restore voltage responses. This indicates that mitochondrial ATP production is not merely recruited downstream of electrical activity; rather, it is permissive for excitability in SA node myocytes. In this view, when the proton-motive force is collapsed, electrical pacing cannot compensate for the energetic requirement needed to sustain membrane responsiveness.

### SA node heterogeneity in the context of entrainment and stochastic resonance

It is important to place our findings in the context of two classical synchronization mechanisms that operate in oscillatory biological networks: entrainment and stochastic resonance. Entrainment refers to the mutual electrotonic coupling of self-oscillating myocytes, whereby the region with the shortest intrinsic cycle length imposes its rhythm on its neighbors, achieving network-wide phase alignment (Jalife, 1984; Anumonwo et al., 1991). This process is highly efficient in well-perfused regions where cells possess comparable, robust metabolic reserves. Stochastic resonance, conversely, is a nonlinear phenomenon in which intrinsic noise enhances the detection and propagation of weak periodic signals (Wiesenfeld and Moss, 1995; Gammaitoni et al., 1998; Hänggi, 2002). Importantly, in the intact SA node, these mechanisms are not mutually exclusive; rather, they coexist and mutually reinforce one another to ensure robust pacemaking. When microvascular rarefaction creates metabolic heterogeneity, the resulting juxtaposition of high-gain and low-gain myocytes harnesses stochastic resonance to optimize network-wide entrainment, preserving overall heart rate frequency and periodicity even as localized pockets of low excitability emerge.

A growing literature demonstrates pronounced electrophysiological heterogeneity along the SA node (Boyett et al., 2000). High-resolution 3D imaging has shown discontinuously and asynchronously propagating Ca^2+^ transients in the intact mouse SA node, arising from spatially heterogeneous local Ca^2+^ release events within the HCN4^+^/Cx43^-^ meshwork (Kim et al., 2018; Monfredi et al., 2018; Bychkov et al., 2020). Complementary work revealed parallel heterogeneity in vascular density, membrane excitability, and Ca^2+^ signaling in myocytes along the longitudinal axis of the node (Grainger et al., 2021). Such variability motivated the hypothesis that intrinsic noise sharpens pacemaker timing via stochastic resonance (Clancy and Santana, 2020; Grainger et al., 2021; Guarina et al., 2022). A recent multiscale study confirmed that this noise fine-tunes SA node firing and preserves rhythmicity under strong parasympathetic drive (Okamura et al., 2024, *Preprint*). Collectively, these data establish microscopic heterogeneity—spanning structure, metabolism, and electrical behavior—as an intrinsic feature of native SA node tissue that critically modulates its function.

Our current findings provide a metabolic explanation for how this variability drives synergistic synchronization: inferior SA node myocytes, constrained by sparse vascular supply and limited mitochondrial reserve, behave as low-frequency, intermittently firing “noise generators.” Notably, the observation that full recovery of mitochondrial ATP dips takes ∼240–282 ms suggests that dip-dominant cells can sustain firing only up to ∼3.5–4.2 Hz without incurring a progressive ATP deficit, which could lead to decreased excitability. Although Mode 2 dips are present in both superior and inferior regions, they predominate in inferior myocytes, meaning that noise generation is not exclusively an inferior-node property but is amplified there by the convergence of sparser vascularization, reduced mitochondrial reserve, and lower intrinsic firing rates. However, rather than representing a simple failure of the network, the variability in ATP-withdrawal and recovery kinetics creates the precise metabolic noise required for stochastic resonance. Subthreshold fluctuations in membrane potential, driven by these deep ATP dips, enhance the sensitivity of the electrotonically coupled network to depolarizing inputs from well-perfused, high-gain cells. In this way, stochastic resonance actively facilitates global entrainment. Our model predicts maximal network coherence when these low-gain, noise-generator cells are coupled to high-gain, well-perfused superior myocytes. This functional synergy aligns with our experimental finding that collapsing the mitochondrial reserve with FCCP or thapsigargin abolishes resonance and the network’s subsequent ability to entrain, underscoring the shared metabolic origin of both processes.

While our experiments were performed in the murine SA node, the bioenergetic machinery we interrogate—mitochondrial Ca^2+^ uptake, tricarboxylic acid cycle activation, and oxidative phosphorylation—is widely conserved. Thus, the electrometabolic insights gained here likely translate broadly to human cardiac physiology. Consistent with this premise, Tagirova Sirenko et al. (2021) demonstrated that the fundamental pacemaking architecture of mouse and human SA node cells is similar. We therefore anticipate that the principle of mutually reinforcing entrainment and stochastic resonance, grounded in metabolic heterogeneity, will apply broadly to other excitable tissues or intrinsic pacemaker networks.

### Capillary–mitochondria–ion channel axis and clinical implications

Crucially, our SA node findings provide direct support for the capillary–mitochondria–ion channel axis recently proposed by our group and colleagues (Santana and Earley, 2026), in which excitability emerges from a coupled system in which capillary density sets the local oxygen ceiling, mitochondrial volume defines energetic bandwidth, and ion channels are tuned by metabolic state. Within this framework, [ATP]_i_ measurements link the superior–inferior energetic hierarchy to underlying vascular and mitochondrial architecture such that higher capillary density and shorter myocyte–capillary distances in the superior node align with higher diastolic–cytosolic [ATP]_i_ (∼0.8 mM), whereas the more sparsely vascularized inferior node exhibits lower [ATP]_i_ (∼0.4 mM) and more oxidized FAD signals, consistent with a chronically supply-limited state. The 0.4–0.8 mM [ATP]_i_ window we document is mechanistically significant because it falls on the steep portion of the ATP dependence for several ion channels and transporters that are central to pacemaking. ATP-sensitive K^+^ (K_ATP_) channels, which can open at [ATP]_i_ values in this range (Inagaki et al., 1995), would progressively activate as [ATP]_i_ falls, generating a hyperpolarizing current that directly opposes diastolic depolarization and reduces firing rate. Simultaneously, RyR2 and SERCA are sensitive to [ATP]_i_ in this concentration range: RyR2 open probability is enhanced by ATP (Kermode et al., 1998; Rios, 2026), such that dips in [ATP]_i_ reduce SR Ca^2+^ release, while reduced SERCA activity impairs SR Ca^2+^ refilling collectively diminishing the Ca^2+^ clock signal that drives pacemaker depolarization. Thus, beat-to-beat ATP dips in Mode 2 cells are not merely energetic footnotes—they constitute a direct feedback signal that transiently suppresses excitability through at least three parallel ion channel and transporter mechanisms. Conversely, the higher [ATP]_i_ sustained in superior, well-perfused cells keeps K_ATP_ channels suppressed, maintains RyR2 open probability and SERCA activity, and thereby supports the robust Ca^2+^ clock signaling and rapid diastolic depolarization characteristic of the dominant pacemaker site.

The clinical relevance of this supply-limited phenotype is underscored by a mouse model of early heart failure with preserved ejection fraction in which selective rarefaction of superior-node capillaries precedes bradycardia and heightened beat-to-beat variability (Manning et al., 2025). We found that rarefaction compromises pacemaker robustness, even when global cardiac output remains normal, indicating that local vascular loss can destabilize rhythm before systemic hemodynamics decline. In the context of this study, however, microvascular rarefaction sculpts pockets of diminished mitochondrial reserve and low excitability, narrowing the roster of regions capable of sustaining high-frequency pacing. When such hypoperfused zones remain interlaced with well-supplied tissue, noise-assisted integration between low-gain and high-gain myocytes can act as a metabolic safety mechanism, injecting timing noise that preserves overall heart rate frequency and beat-to-beat regularity despite localized deficits. Thus, while the leading pacemaker remains mobile, its performance—and resilience—is ultimately governed by the interplay among vascular architecture, mitochondrial content, and microdomain ATP dynamics. The MCU–ANT axis we uncover here operationalizes this paycheck-to-paycheck logic: each Ca^2+^ transient triggers a burst of oxidative phosphorylation that depends on Ca^2+^ entry into the matrix and on rapid ADP/ATP exchange across the inner membrane.

### Overall model and conclusion

In summary, our work extends a unified paycheck-to-paycheck model of cardiac energetics from ventricular myocytes to the pacemaker SA node. In this view, every heartbeat is supported by ATP that is generated, used, and renewed on a cycle-by-cycle basis: ionic and Ca^2+^ clocks set the rate at which ATP is produced; SR-to-mitochondrial Ca^2+^ transfer generates ATP via oxidative phosphorylation; and local vascular supply determines the O_2_ supply needed to generate it. Whether a cell initiates the rhythm or follows it, its electrical and mechanical output is underwritten by the same just-in-time metabolic calculus. Variations in capillary density or mitochondrial content therefore shape not only contractile power but also the locus and stability of pacemaking. Recognizing that the heart is an organ that functions on the capacity of each beat to generate the ATP it needs for excitation–contraction coupling provides a common framework for interpreting how supply–demand mismatches give rise to arrhythmias, bradycardia, and pump failure across diverse physiological and pathological settings.

## Data availability

The data files supporting all findings presented in this paper are available from the corresponding author.
